# The potential of COVID-19 patients’ sera to cause antibody-dependent enhancement of infection and IL-6 production

**DOI:** 10.1038/s41598-021-03273-0

**Published:** 2021-12-09

**Authors:** Jun Shimizu, Tadahiro Sasaki, Atsushi Yamanaka, Yoko Ichihara, Ritsuko Koketsu, Yoshihiro Samune, Pedro Cruz, Kei Sato, Naomi Tanga, Yuka Yoshimura, Ami Murakami, Misuzu Yamada, Kiyoe Itoi, Emi E. Nakayama, Kazuo Miyazaki, Tatsuo Shioda

**Affiliations:** 1 MiCAN Technologies Inc., KKVP 1-36, Goryo-ohara, Nishikyo-Ku, Kyoto, 615-8245 Japan; 2 Department of Viral Infection, Research Institute for Microbial Diseases, Osaka University, 3-1, Yamada-oka, Suita, Osaka 565-0871 Japan; 3 Faculty of Tropical Medicine, Mahidol-Osaka Center for Infectious Diseases, Mahidol University, Bangkok, Thailand

**Keywords:** SARS-CoV-2, Infection

## Abstract

Since the emergence of severe acute respiratory syndrome coronavirus 2 (SARS-CoV-2), many vaccine trials have been initiated. An important goal of vaccination is the development of neutralizing antibody (Ab) against SARS-CoV-2. However, the possible induction of antibody-dependent enhancement (ADE) of infection, which is known for other coronaviruses and dengue virus infections, is a particular concern in vaccine development. Here, we demonstrated that human iPS cell-derived, immortalized, and ACE2- and TMPRSS2-expressing myeloid cell lines are useful as host cells for SARS-CoV-2 infection. The established cell lines were cloned and screened based on their function in terms of susceptibility to SARS-CoV-2-infection or IL-6 productivity. Using the resulting K-ML2 (AT) clone 35 for SARS-CoV-2-infection or its subclone 35–40 for IL-6 productivity, it was possible to evaluate the potential of sera from severe COVID-19 patients to cause ADE and to stimulate IL-6 production upon infection with SARS-CoV-2.

## Introduction

Antibody-dependent enhancement (ADE) of infection has been reported for several virus infections such as dengue^1,2^ and Zika viruses^3^; severe acute respiratory syndrome (SARS)^4^ and Middle East respiratory syndrome (MERS) coronaviruses^5^; human immunodeficiency virus (HIV)^6^; and Ebola virus^7^. In addition, ADE has also been reported in several virus vaccination trials and has become a major concern in vaccine development^8–10^. ADE, particularly that of coronavirus infection, occurs through Fc receptor (FcR)-mediated internalization of the virus and anti-virus Ab complex into FcR-expressing immune cells such as myeloid cells and B cells^4,5,11–13^. Furthermore, recent studies have demonstrated that human myeloid cells including tissue-resident alveolar macrophages, monocytes derived from peripheral blood mononuclear cells, and myeloid cell lines can be infected with SARS-CoV-2^14–17^, although it is not clear whether this infection in myeloid cells is productive or not. Taken together, these reports suggest the involvement of myeloid cells in possible ADE even in SARS-CoV-2 infection. However, in the case of SARS-CoV-2, ADE has yet to be evaluated in immune cells for infection with live authentic SARS-CoV-2^4,15^.

In this study, to establish a system to evaluate ADE in FcR-expressing immune cells using live SARS-CoV-2, we used our platform to generate immortalized myeloid cell lines (referred to as Mylc lines, Supplemental Fig. S1) from human iPS cells^18–20^ and asked whether Mylc lines can be infected and are available for the detection of ADE. We show that using Mylc lines, it is possible to detect the potential of sera from COVID-19 patients to enhance infection of SARS-CoV-2 and to augment IL-6 production. The elevated production of inflammatory cytokines including IL-6, so called cytokine storms, has been reported in severe acute respiratory distress syndrome in COVID-19^21,22^. Therefore, Mylc lines can be used to analyze two dangerous adverse effects, the enhancement of virus infection and the augmentation of IL-6 production, present in the sera of COVID-19 patients.

## Results and discussion

**Figure 1:**
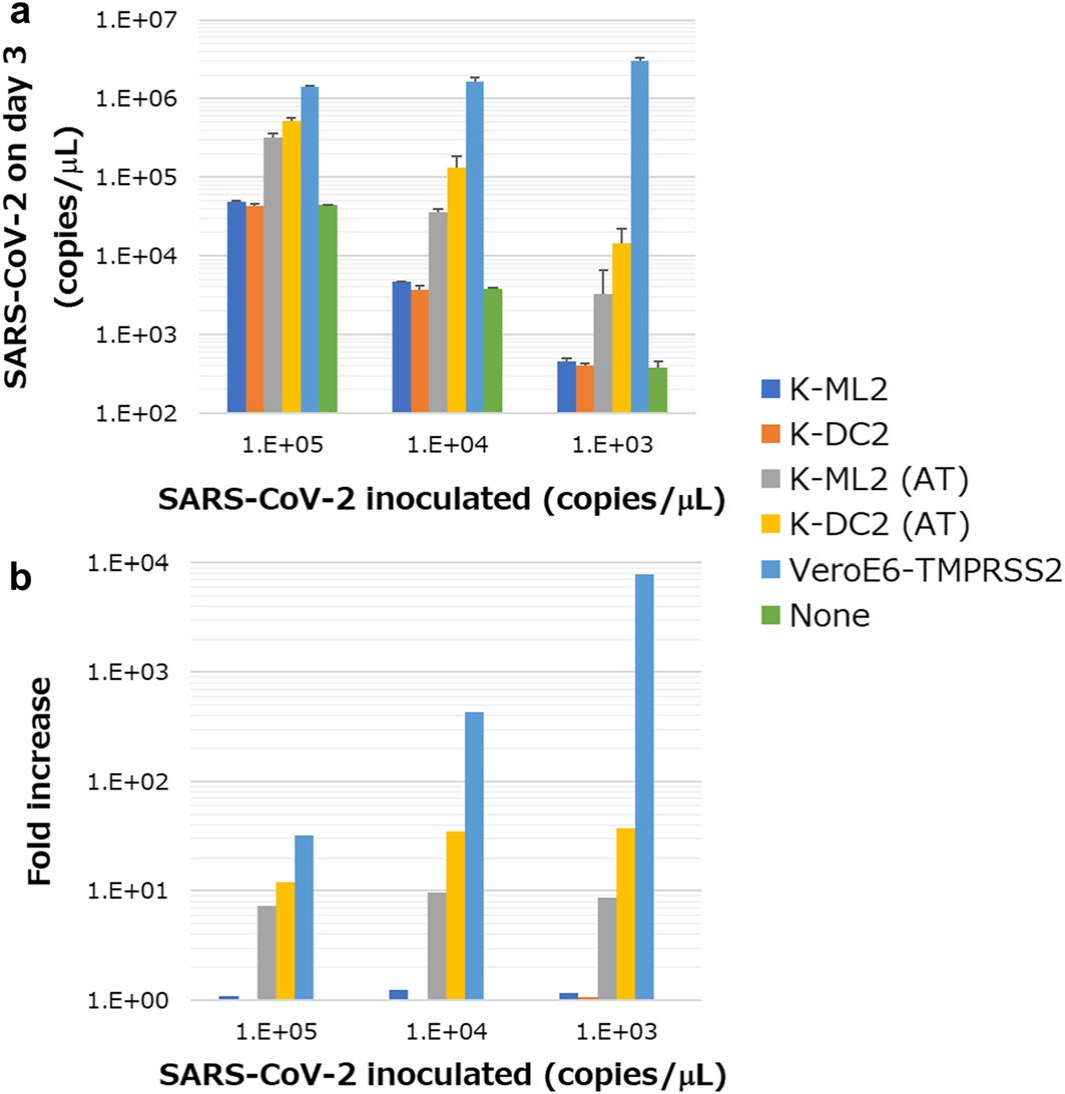
Infection of SARS-CoV-2 in Mylc cell lines. K-ML2 and K-ML2 (AT) cells before and after DC-differentiation (2 × 10^4^/well in a 96-well flat plate) were co-cultured with three different doses of SARS-CoV-2. As a positive control, VeroE6 cells expressing TMPRSS2 (VeroE6-TMPRSS2, 1 × 10^4^/well) were used. As a negative control, viruses were cultured in the absence of any cells (indicated as None). (**a**) Three days later, the amount of virus in culture SNs was measured by qPCR. (**b**) The increment of viruses is expressed as the fold increase compared with the amount of viruses cultured in the absence of cells for 3 days. Error bars indicate SD. N = 3.

Using our platform, several Mylc lines have been established from different human iPS cells or different PBMC donors and they can be infected with dengue virus. Their infectibility is comparable to that of Vero cells (Supplemental Fig. S2). In the present study, we used the Mylc line K-ML2 (Supplemental Fig. S1), because this line grows fast and it is easy to prepare a large number of cells. First, we examined whether K-ML2 cells can be infected with live SARS-CoV-2 (Fig. 1). K-ML2 cells were inoculated with serially-diluted SARS-CoV-2 and cultured for three days. Contrary to our expectation, no increment of SARS-CoV-2 in the culture supernatants (SNs) was detected by quantitative PCR (qPCR). K-ML2 cells can be differentiated into dendritic cells (DCs) by culturing them with IL-4 for three days. The resulting differentiated K-ML2 cells, hereafter called K-DC2 cells, expressed lower amounts of CD14 than undifferentiated K-ML2 cells did^23^ (Supplemental Fig. S3a). Even after differentiation into DCs, K-DC2 cells were still not susceptible to SARS-CoV-2 replication (Fig. 1).

Angiotensin-converting enzyme 2 (ACE2) is the binding receptor of SARS-CoV-2, and transmembrane protease serine 2 (TMPRSS2) processes the spike glycoprotein of SARS-CoV-2 for membrane fusion between the virus envelope and cellular membrane of target cells^24^. We next investigated whether K-ML2 cells express these molecules. qPCR experiments revealed that they express these molecules at almost basal levels (Supplemental Fig. S4). We therefore prepared ACE2- and TMPRSS2-expressing K-ML2 cells (K-ML2 (AT) cells, Supplemental Fig. S1) using a lentivirus expression system. The levels of expression of ACE2 and TMPRSS2 in K-ML2 (AT) cells were augmented more than 100 times (Supplemental Fig. S4) after lentivirus transduction. We then inoculated live SARS-CoV-2 into K-ML2 (AT) cells before and after DC differentiation and examined the levels of SARS-CoV-2 RNA in the culture SNs of K-ML2 (AT) or K-DC2 (AT) cells by qPCR three days after inoculation (Fig. 1). We observed an approximately 30-fold increase of SARS-CoV-2 RNA compared with the control culture (the inoculated viruses alone without cells). These results suggested that K-ML2 (AT) cells, especially after DC-differentiation (K-DC2 (AT)), can be used as host cells for SARS-CoV-2 infection. Microscopic observation of SARS-CoV-2-infected K-DC2(AT) cells at three days after infection revealed virtually no cytopathic effects (Supplemental Fig. S5a,b). In both uninfected (Supplemental Fig. S5a) and infected cells (Supplemental Fig. S5b), approximately 90% of cells were round-shaped and floating, while the remaining cells adhered to the plate.

In the experiment shown in Fig. 1, the inoculated viruses were not washed out during three days’ culture. In the second protocol, K-DC2 (AT) cells were mixed with viruses in tubes, incubated for 4 h, washed once, and then seeded in 96-well culture plates (Supplemental Fig. S6b). In the third protocol, K-DC2 (AT) cells were mixed with viruses in 96-well culture plates, and the culture SNs were removed as much as possible after four hours of incubation. Fresh medium was then added to the cells, and cells were cultured for 3 days (Supplemental Fig. S6c). In these two protocols, a lower background in the control culture and higher fold-increase of viruses in SNs from the infected K-DC2 (AT) cells were observed, ranging from a more than 100-fold to an approximately 1000-fold increase, compared to those without the washing step (Supplemental Fig. S6a). It is noteworthy that after washing away free viruses, the expansion of the viruses was not observed at the lowest doses of virus inoculation (1000 copies/μL; see Supplemental Fig. S6b,c). In the protocol without washing (Fig. 1, Supplemental Fig. S6a), we observed an increase of viruses at the same dose of virus inoculation. These results suggested that the attachment of SARS-CoV-2 to K-DC2 (AT) cells is relatively slow.

**Figure 2:**
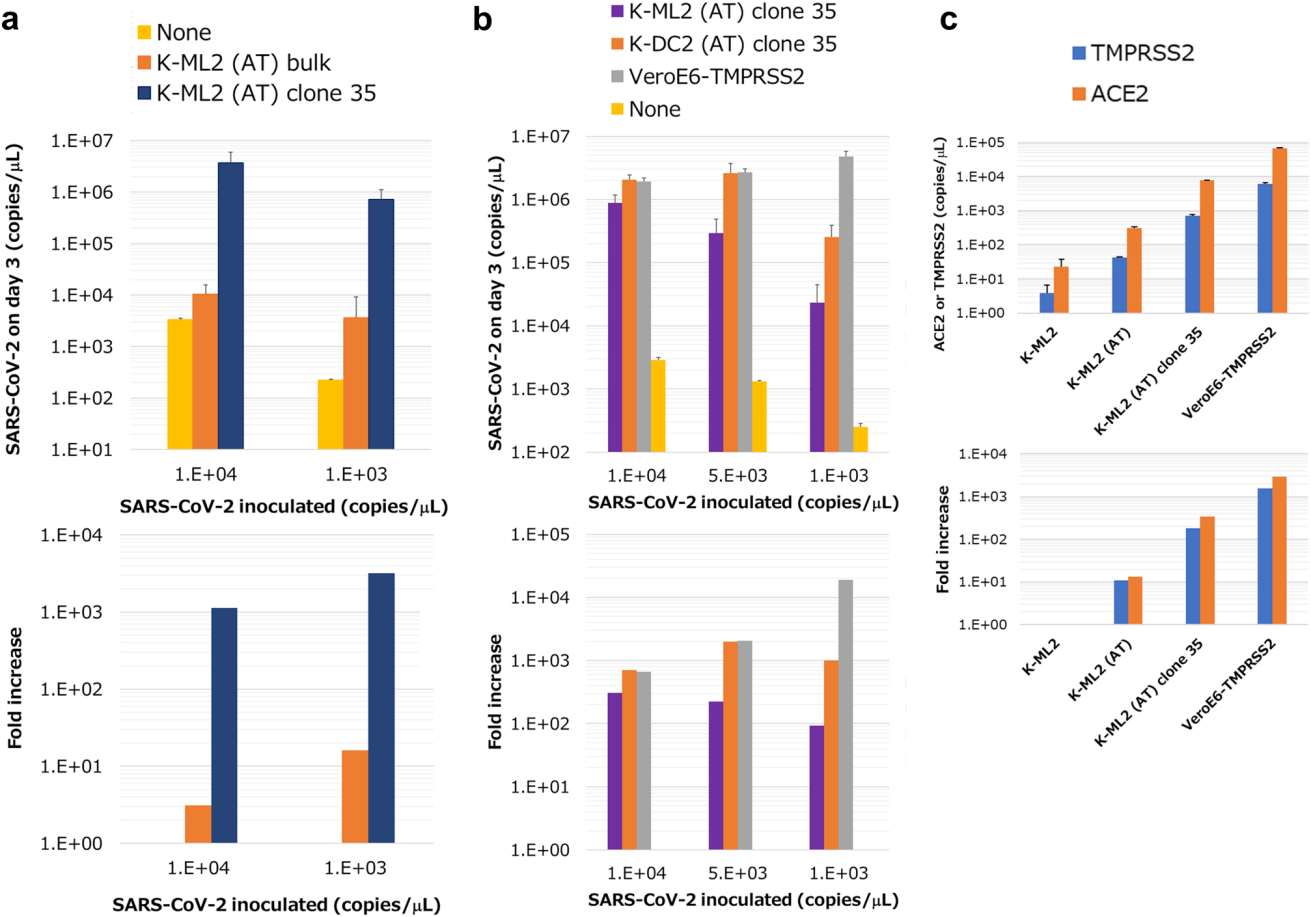
Comparison of K-ML2 (AT) bulk and K-ML2 (AT) clone 35. (**a**) K-ML2 (AT) bulk and clone 35 cells or (**b**) clone 35 cells before and after DC-differentiation were cultured with SARS-CoV-2 and evaluated as in Fig. 1. (**c**) The amount of ACE2 and TMPRSS2 in the indicated cells was measured by qPCR (top). The fold increase compared with K-ML2 is shown in the bottom panel. Error bars indicate SD. N = 3.

K-ML2 (AT) cells were supposedly a mixture of numerous clones when we introduced ACE2 and TMPRSS2 genes. We therefore established K-ML2 (AT) clones by limiting dilution. The obtained clones were screened and selected based on their infectibility. The resulting clone, clone 35 (Supplemental Fig. S1), was significantly more sensitive to infection with SARS-CoV-2, even before DC-differentiation (Fig. 2a), than the parental cell mixture. The differentiation of clone 35 cells into DCs resulted in the down-expression of CD14 (Supplemental Fig. S3b) and higher levels of virus production in SNs (Fig. 2b). Furthermore, the levels of virus production of clone 35 cells were comparable to those of VeroE6-TMPRSS2 cells when more than 5000 copies/μL of virus were inoculated (Fig. 2b). qPCR analysis revealed that clone 35 cells expressed ACE2 and TMPRSS2 at an approximately tenfold higher level than parental K-ML2 (AT) cells, but still tenfold lower than VeroE6-TMPRSS2 (Fig. 2c). We could not observe obvious cytopathic effects even in clone 35 cells (Supplemental Fig. S5c,d). Taken together, these results demonstrated that K-ML2 (AT) clone 35 or parental cells can be used in studying the infection of human leukocytes with live SARS-CoV-2.

It is known that myeloid cells express FcRs. In fact, other Mylc lines established by the same methods express FcγRI, II and III^25^. We also confirmed the expression of FcR on K-ML2 cells by functional evaluation. K-ML2 cells are a myeloid cell line and do not express T cell receptor (TCR). However, K-ML2 cells can be stained with the anti-human TCR Ab (mouse IgG_2a_). This staining is abolished by an Fc-blocker, demonstrating the existence of functional FcR on K-ML2 cells (Supplemental Fig. S7). It has been demonstrated that FcR-mediated uptake of the virus and anti-virus Ab complex is one mechanism of ADE in dengue viruses^1,26–28^. Furthermore, parental K-ML2 (AT) cells and cloned cells could be infected with SARS-CoV-2 (Figs. 1, 2) and express FcR (Supplemental Fig. S7). These results prompted us to examine whether we could observe ADE of SARS-CoV-2 in K-ML2 (AT) cells. First, we examined whether serum prepared from SARS-CoV-2-immunized mice has the potential to induce ADE (Supplemental Fig. S8). Serially-diluted serum specimens from immunized mice were pre-mixed with a constant dose of SARS-CoV-2 (1250 copies/μL), and K-ML2 (AT) clone 35 cells were added to the mixture. Three days later, the levels of viruses in SNs were quantitated by qPCR. Serum from immunized mice indeed augmented SARS-CoV-2 replication by 5 and 8 times when diluted 10^–4^- and 10^–5^-fold, respectively (Supplemental Fig. S8b). At higher concentrations of immunized serum, neutralizing activity was also observed (Supplemental Fig. S8c), since virus replication was suppressed to less than 1% of the control without immunized serum when diluted 10^–3^- or 10^–2^-fold. Normal mouse serum showed no ADE activity under the same experimental conditions (Supplemental Fig. S8a). These results demonstrated that K-ML2 (AT) clone 35 cells can be used to detect the ADE-causing potential of anti-SARS-CoV-2 Abs.

**Figure 3:**
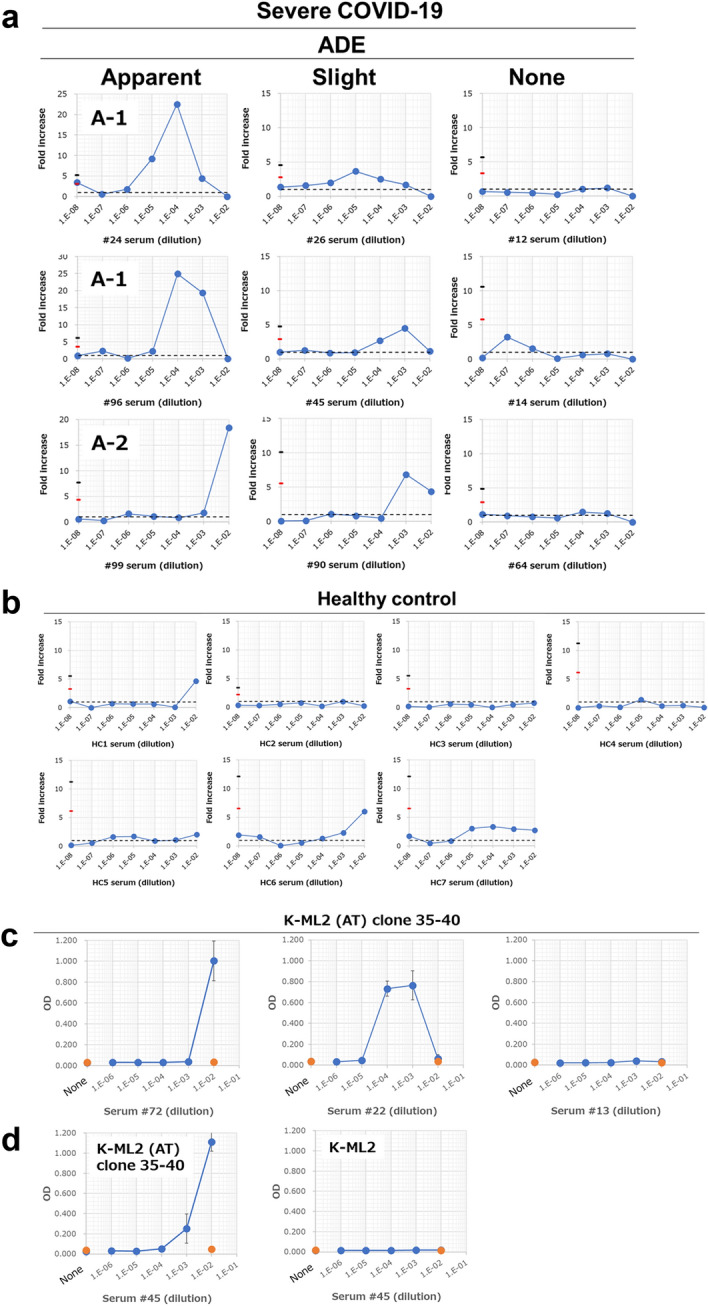
Infection- and IL-6 production-enhancing activity of sera derived from patients infected with SARS-CoV-2. K-ML2 (AT) clone 35 cells (2 × 10^4^/well in a 96-well flat plate) were cultured with SARS-CoV-2 (1.25 × 10^3^ copies/μL) in the presence (N = 3) or absence (N = 6–9, control culture) of serially-diluted serum from (**a**) COVID-19 patients (N = 100) or (**b**) healthy volunteers (N = 7). The fold increase of virus progeny in SNs is shown. Red or black lines on the y-axis indicate the mean of control culture + a three or six SD cut-off, respectively. In each qRT-PCR experiment, the background control cultures (N = 6–9) were set. The raw data in each qRT-PCR experiment are summarized in Supplemental Table S3. Therefore, the values for red and black lines are different in each graph. The black dotted line indicates fold increase = 1. The results from COVID-19 patients were classified into three groups (Apparent, Slight, and None). Three representative examples in each group are shown in (**a**). All results from COVID-19 patients are shown in Supplemental Fig. S9. Patients of the Apparent group were further classified into two subgroups, A-1 and A-2, as indicated in (**a**). (**c**) K-ML2 (AT) clone 35–40 cells (2 × 10^4^/96-well flat plate) were cultured with (blue circles) or without (orange circles) SARS-CoV-2 (1 × 10^4^ copies/μL) in the presence or absence of serially-diluted serum from COVID-19 patients. Three days later, the amount of IL-6 in culture SNs was measured by ELISA. The amount of IL-6 is plotted as OD values. Error bars indicate SD. N = 3. (**d**) K-ML2 (AT) clone 35–40 cells and parental K-ML2 cells were cultured as in (**c**) with the same serum #45. The production of IL-6 was measured (N = 3).

Based on these data, we next examined whether sera derived from patients with severe COVID-19 (N = 100, Supplemental Table S1) had the potential to induce ADE. In these experiments, we used serially-diluted serum, a constant dose of virus (1,250 copies/μL), and K-ML2 (AT) clone 35 cells. Figure 3a and Supplemental Figs. S9-1–9 demonstrate that nearly half (49 out of 100 samples, Table 1) of serum samples from COVID-19 patients examined had the ability to cause ADE to a greater or lesser degree at different Ab concentrations. Experiments using representative ADE-causing sera demonstrated that ADE depends on FcR, because the pre-existence of FcR-bindable irrelevant Ab (4G2) can inhibit ADE (Supplemental Fig. S10). We analyzed individual results (N = 100) in ADE assays from two aspects: the magnitude of the ADE peak and the antibody titer that causes ADE. First, for the magnitude of ADE, we used two cut-off values (mean + 3 × SD and mean + 6 × SD) to classify these individual results into three groups: apparent ADE, slight ADE, and no ADE (indicated as None). Details of the definitions of these groups are described in ADE assay section of the Methods. As summarized in Table 1, 27 out of 100 samples (27.0%) showed apparent ADE, 22/100 (22.0%) slight ADE, and 51/100 (51.0%) no ADE. We did not observe any differences between male- and female-derived samples (Table 1). We observed an increasing tendency of anti-SARS-CoV-2 Ab quantity (median in Supplemental Table S2) in apparent and slight ADE groups compared with the no ADE group, although these differences did not reach statistical significance. Second, the apparent ADE group was further classified into two subgroups based on the Ab titer that caused ADE (see Fig. 3a): the first subgroup (A-1) showed ADE peaks at high dilutions, i.e., at a lower concentration of serum (for example, sera #24 and #96 shown in Fig. 3a, N = 21); and the second subgroup (A-2) showed ADE activity at the highest serum concentration examined (serum #99 shown in Fig. 3a,  N = 6). There was no difference in the amount of anti-SARS-CoV-2 IgG between A-1 and A-2 (median: 2.69 (N = 21) and 2.56 (N = 6), respectively). As a control, sera examined from healthy donors (N = 7) showed no apparent ADE activity (Fig. 3b). Unfortunately, clinical information of the samples is limited, and no further correlation of observed ADE with clinical information can be analyzed at this moment. It is necessary to analyze the ADE activity of sera from various clinical manifestations of COVID-19. It is also necessary to analyze the change in ADE activity during the course of infection with SARS-CoV-2. Nevertheless, it is possible that the A-2 subgroup represents a more dangerous pattern of Ab-mediated response against SARS-CoV-2, because ADE would be more dominant than neutralizing activity in A-2. Therefore, pre-screening of patient’s serum to find a pattern that could be categorized into A-2 may be helpful in predicting clinical outcomes during the course of infection.

**Table 1:** ADE activity in sera from severe COVID-19 patients.

Information	Severe COVID-19			
	Total	ADE		
		Apparent	Sight	None
Patient no. (%)	100 (100)	27 (27.0)	22 (22.0)	51 (51.0)
Male no. (%)	59 (100)	16 (27.1)	13 (22.0)	30 (50.8)
Female no. (%)	41 (100)	11 (26.8)	9 (22.0)	21 (51.2)

Information about ADE activity in each serum sample is derived from Fig. 3a and Supplemental Fig. S9. The numbers and percentages in each category are indicated.

The elevated production of inflammatory cytokines (a cytokine storm, one of the most dangerous consequences of SARS-CoV-2 infection) has been reported in severe cases^29–32^, for example acute respiratory distress syndrome (ARDS), in COVID-19^21,22,33–35^. Among numerous cytokines associated with the ARDS, IL-6 is particularly important in the persistence of the proinflammatory milieu^36–38^. In concert, it has also been reported that IL-6 levels are significantly elevated and associated with adverse clinical outcomes in COVID-19 patients^39^, although the effect of anti-IL-6 Abs is limited. Myeloid cells are an inflammatory cytokine-producing cell source^17^. This information prompted us to ask whether the myeloid cell line K-ML2 (AT) can produce IL-6, as one example of a major inflammatory cytokine, upon stimulation with live SARS-CoV-2. To this end, K-ML2 (AT) clone 35 cells were cultured with a titrated amount of SARS-CoV-2 in the presence or absence of ADE-causing serum #73 (belonging to the A-1 subgroup) for 3 days (top panels in Supplemental Fig. S11). Interestingly, we observed IL-6 production only in the presence of SARS-CoV-2 and serum #73, although a high dose of virus (10^5^ copies/μL) was required for IL-6 production. Viruses alone had no effect on the IL-6 production even with a dose of 10^5^ copies/μL. To more sensitively detect IL-6, we re-cloned K-ML2 (AT) clone 35 cells and screened clones based on their potential to produce IL-6 upon stimulation with SARS-CoV-2 (Supplemental Fig. S1). The results of three representative clones, clones 35-3, 35-20, and 35-40, are shown in Supplemental Fig. S12a. Even after re-cloning, a high dose of virus was still required for the production of IL-6. However, these re-cloned cells exhibited higher production of IL-6 in the presence of viruses and ADE-causing serum (serum #32 shown in Supplemental Fig. S12a and serum #73 in Supplemental Fig. S11, bottom panels) than clone 35 cells did. We then used clone 35–40 for further analysis. The addition of COVID-19 patient serum or serum from SARS-CoV-2-immunized mice to a culture of clone 35–40 cells together with live SARS-CoV-2 induced the production of IL-6 (Fig. 3c, Supplemental Figs. S12, S13-1–9). Seventy out of 100 serum samples of SARS-CoV-2-infected individuals exhibited IL-6 production more than OD ≥ 0.4 (Table 2). The blockade of FcR resulted in the reduced production of IL-6, demonstrating FcR dependency in the enhancement of IL-6 production by COVID-19 patient serum (Supplemental Fig. S14). It should be emphasized that ADE-causing sera do not always cause enhanced IL-6 production (Table 2), suggesting the limitation of IL-6 as a parameter and the requirement of other inflammatory cytokine parameters including IL-8 and TNF-α; however, ADE-causing sera exhibited higher induced IL-6 production (IL-6 induction-positive (OD ≥ 0.4): 88.9% in Apparent, 72.7% in Slight, and 58.8% in None groups, and notably 100% in the A-2 subgroup) (Table 2). Among these categorized groups based on ADE activity and IL-6-enhancing activity, there was no significant difference in the amount of anti-SARS-CoV-2 IgG. Augmented IL-6 production in the presence of COVID-19 patient serum was not observed in parental K-ML2 cells (Fig. 3d), suggesting the requirement of ACE2 and TMPRSS2 for IL-6 production. The ADE-causing serum alone and serum from healthy donors had no induced IL-6 production (Fig. 3c, Supplemental Figs. S12b, S13). Taken together, these results demonstrated that K-ML2 (AT) clone 35 and clone 35–40 cells can be used to analyze two dangerous adverse effects, the enhancement of virus infection (using clone 35 cells) and the augmentation of IL-6 production (using clone 35–40 cells), present in the sera of COVID-19 patients. The detection of serum augmentation of IL-6 production might be helpful in understanding and predicting cytokine storms in the disease process of COVID-19.

**Table 2:** ADE activity and the potential to enhance IL-6 production of sera from COVID-19 patients.

Severe COVID-19					
ADE category	No.			Mean (anti-SARS-CoV-2 IgG)	
	Total	IL-6 (OD)		IL-6 (OD)	
		≧ 0.4	< 0.4	≧ 0.4	< 0.4
Whole	100	70 (70.0)	30 (30.0)	2.27	2.34
Apparent	27	24 (88.9)	3 (11.1)	2.54	2.43
A-1	21	18 (85.7)	3 (14.3)	2.53	2.43
A-2	6	6 (100.0)	0 (0.0)	2.58	
Slight	22	16 (72.7)	6 (27.3)	2.34	2.09
None	51	30 (58.8)	21 (41.2)	2.01	2.40

Based on the results from Fig. 3a and Supplemental Figs. S9 and S13, each serum sample from COVID-19 patients (N = 100) was divided into several groups as shown. More than 0.4 of the top OD value in the ELISA assay was regarded as positive activity to enhance IL-6 production. The number in parentheses means the percentage in each ADE category.

In the present study, we showed that a human myeloid cell line (K-ML2 (AT) cells) could be infected with SARS-CoV-2 only after introducing ACE2 and TMPRSS2 into the parental K-ML2 cells (Fig. 1). Furthermore, the efficiency of infection was dependent on the amount of these molecules (Fig. 2, Supplemental Fig. S4). In accordance with these results, findings to date indicate that myeloid cells are not the major hosts in SARS-CoV-2 infection in terms of susceptibility and their expression of critical molecules such as ACE2^11,12,15^, although some reports suggest myeloid cells as important players in the immune response against SARS-CoV-2^14^. However, it is plausible that myeloid cells express ACE2 at low levels^40^, and it can be upregulated by cytokines^41^. It is also possible that co-expression of ACE2, TMPRSS2, and FcR in the same myeloid cells might result in ADE at the single cell level. In the case of the alveolus, where ACE2-highly expressing alveolar cells and tissue-resident alveolar macrophages coexist, the presence of ADE-inducible myeloid cells might lead to acute respiratory disorders, and myeloid cell lines established in this study will be helpful in studying this possibility in lung tissues.

The development of vaccines against SARS-CoV-2 is urgently needed^11^. After vaccination, the appearance and duration of neutralizing Abs would be the major achievement. However, at the same time, we also have to pay attention to the ADE phenomenon that may be caused by Abs^11,12^. Recently, an experimental model using a pseudo-virus of SARS-CoV-2 demonstrated ADE by monoclonal antibodies or plasma of patients with COVID-19 who recovered^42–47^. In contrast, in the present study, we showed that a human leukocyte cell line (K-ML2 (AT) clone 35) can itself be used as a host for infection with live SARS-CoV-2. Furthermore, we demonstrated that K-ML2 (AT) clone 35 cells are useful to detect ADE activity by Abs. These data suggest that K-ML2 (AT) clone 35 cells can serve in monitoring the ability of Abs (neutralizing or ADE-causing) induced by vaccine candidates.

ADE caused by dengue virus results in a massive augmentation of virus production ranging from 1000- to 10,000-fold enhancement in general^2^. In contrast, ADE of SARS-CoV-2 demonstrated in the present study resulted in at most a tenfold increment in the majority of patients, except for patient serum #94 (Supplemental Fig. S9-3). Optimistically, ADE in SARS-CoV-2 may not be a major concern at this moment. However, in Manaus, Brazil, a large re-expansion of COVID-19 was recently reported even in a population with more than 75% seroprevalence of SARS-CoV-2^48^. One possible reason for this resurgence was early decline of humoral immunity against SARS-CoV-2^49–53^. Alternatively, escape variants from the acquired immunity might have emerged. In any case, it is possible that ADE might have played a major role in this SARS-CoV-2 resurgence in Manaus. Furthermore, in the future when SARS-CoV-2 diverges into several different serotypes like dengue virus has, ADE in SARS-CoV-2 will become a more important concern. It is thus important to keep monitoring the ADE- and IL6-inducing activities of COVID-19 patient sera using several divergent SARS-CoV-2 strains^54–56^ to see whether more massive augmentation of virus production and/or IL6 induction can be observed.

## Methods

### Establishment of Mylc cell lines

The procedure to generate Mylc lines was previously reported^18–20^. Briefly, immortalized myeloid cell lines were established by the lentivirus-mediated transduction of cMYC, BMI-1, GM-CSF, and M-CSF into human iPS cell-derived myeloid cells (obtained from Dr. Satoru Senju (Kumamoto University, Japan), Supplemental Fig. S1)^18–20^. These cell lines were further induced to express ACE2 and TMPRSS2 using lentiviral vectors. The established cell lines were maintained as bulk cell lines. In some experiments, cloned cells established from bulk lines by limiting dilution were used. These cloned cells were stable in terms of function (susceptibility to infection and IL-6 productivity) at least for 5 months. These clones were maintained at longest for two months and used for experiments. The expression of each molecule (ACE2 and TMPRSS2) at the RNA level was confirmed as follows: total RNA was extracted from cells and purified with TRIzol Reagent (Invitrogen) and Deoxyribonuclease (RT Grade) for Heat Stop (Nippon Gene) according to the manufacturer’s protocol. Total RNA was reverse transcribed by the SuperScript III First-Strand Synthesis System for RT-PCR (Invitrogen) following the manufacturer’s instructions. The primers used for qRT-PCR were as follows: ACE2 forward, GGGATCAGAGATCGGAAGAAGAAA, reverse, AGGAGGTCTGAACATCATCAGTG; TMPRSS2 forward, AATCGGTGTGTTCGCCTCTAC, reverse, CGTAGTTCTCGTTCCAGTCGT. In some experiments, these cell lines were cultured in the presence of IL-4 (100 ng/mL) for three days to differentiate into dendritic cells. Each cell line was cultured in MEM alpha (Gibco) supplemented with 10% (vol/vol) fetal bovine serum under 37 °C, 5% CO_2_, and water-saturated humidity conditions.

### Immunostaining

For flow cytometry, cells were resuspended in Fc blocking buffer (1:500 dilution, eBioscience) and incubated on ice. Twenty minutes later, without washing, cells were stained with the following Abs: anti-CD14-FITC or FITC-conjugated isotype control Ab (all purchased from BioLegend). Stained cells were analyzed by flow cytometry.

### Viruses

SARS-CoV-2/Hu/DP/Kng/19-020 (GenBank accession number: LC528232.1) was provided from Kanagawa Prefectural Institute of Public Health, Kanagawa, Japan. SARS-CoV-2 viruses were passaged in VeroE6-TMPRSS2 cells (purchased from National Institutes of Biomedical Innovation, Health and Nutrition, JCRB cell bank, Japan) two times. Virus stock was aliquoted, examined for the presence of virus RNA by quantitative RT-PCR, and tested for mycoplasma (the result was mycoplasma-free) before being used for experiments. All experiments using live SARS-CoV-2 followed the approved operating procedures of the biosafety level 3 facility at the Research Institute for Microbial Diseases of Osaka University.

### In vitro infection of cells

Mylc cell lines (2 × 10^4^/well) or VeroE6-TMPRSS2 cells (1 × 10^4^/well) were cultured with titrated amounts of SARS-CoV-2 virus in 96-well flat-bottomed plates. Viral concentrations in culture SNs after 3 days of culture were determined by quantitative RT-PCR. Total RNA was extracted from culture SNs using the QIAamp^®^ viral RNA mini kit (QIAGEN) following the manufacturer’s instructions. The primers used for qRT-PCR were as follows: SARS-CoV-2 forward, AGCCTCTTCTCGTTCCTCATCAC, reverse, CCGCCATTGCCAGCCATTC. The fold increases in viral quantity were calculated as follow: fold increase = (virus concentration (copies/μL) of experimental group)/(virus concentration (copies/μL) of virus alone without cells, cultured for the same period).

### Serum from mice immunized with SARS-CoV-2

Anti-SARS-CoV-2 mouse serum was obtained from BALB/c mice immunized with formaldehyde-inactivated SARS-CoV-2 three times. Each immunization was performed every 2 weeks, first with a complete adjuvant, second with an incomplete adjuvant, and third without an adjuvant. Three days after the last immunization, whole blood was collected from the mice, and serum was separated from the blood. It was confirmed that the serum from immunized mice reacted with Vero cells infected with SARS-CoV-2. This animal experiment was approved by the Institutional Committee of Laboratory Animal Experimentation (Research Institute for Microbial Diseases, Osaka University. Project number: R01-17-1). All procedures in this animal experiment were conducted in a manner to avoid or minimize discomfort, distress or pain to the animals according to ARRIVE guidelines (https://arriveguidelines.org/), and the approved operating procedures and guidelines at the Research Institute for Microbial Diseases of Osaka University.

### ADE assay

Serum samples from uninfected donors were obtained from volunteers. Informed consent was obtained from all subjects involved in this study. The Institutional Review Board of the Research Institute for Microbial Diseases, Osaka University (approval number: 31-14) approved collection and use of sera from uninfected volunteers in this study. We confirmed that all healthy donors had no Abs binding or neutralizing to SARS-CoV-2 in other experiments. Serum samples from COVID-19 patients, who had been transferred to the ICU before, were purchased from REPROCELL (Kanagawa, Japan). Informed consent was obtained from all subjects. All experiments using materials from human followed the approved operating procedures and guidelines at the Research Institute for Microbial Diseases of Osaka University. The amount of anti-SARS-CoV-2 IgG in each serum sample was measured using the VITROS™ Anti-SARS-CoV-2 Total Reagent Pack and attached as sample information (REPROCELL, Kanagawa, Japan). In some experiments, serum from mice immunized with SARS-CoV-2 was also used. These sera were used after heat inactivation at 56 °C for 30 min. In ADE assays, viruses were first mixed with serially-diluted serum for 10 min at 37 °C, then cells were added to the mixtures. Cells cultured along with viruses in the absence of Abs were used as a background control. The fold increases in viral quantity were calculated as follows: the fold increase = (virus concentration (copies/μL) of the experimental group with Ab)/(virus concentration (copies/μL) of the background control culture). The cut-off values for the enhancement of activity were calculated from the means + three or six standard deviations (SD) in the amounts of viruses (copies/μL) obtained with six to nine background control cultures in each qRT-PCR experiment. Using these cut-off values, serum samples were classified into three groups as follows: Apparent ADE = the top value of fold increase was more than the mean + six SD of control cultures; Slight ADE = the top value of fold increase was between the mean + three and six SD of control cultures; None = the top value of fold increase was under the mean + three SD of control cultures.

### IL-6 measurement

Mylc cell lines (2 × 10^4^/well) were cultured for three days with SARS-CoV-2 viruses in the presence or absence of serum in 96-well flat-bottomed plates. Three days later, SNs were harvested and UV irradiated. The amount of IL-6 in SNs was measured by an enzyme linked immunoassay (BioLegend) and plotted as OD values.

### Statistical analysis

The statistical significance of differences was evaluated with the Mann–Whitney test. Probability (p) less than 0.05 was considered significant.

## Supplementary Information

Supplementary Information.
