# Supplementary material for: The potential of COVID-19 patients’ sera to cause antibody-dependent enhancement of infection and IL-6 production
Source: Sci Rep. 2021 Dec 9;11:23713. doi: 10.1038/s41598-021-03273-0 (PMC8660863; doi:10.1038/s41598-021-03273-0)
Supplement: Supplementary file 1 — Supplementary Information. [file 41598_2021_3273_MOESM1_ESM.pdf]

Supplemental information

The potential of COVID-19 patients' sera to cause antibody-dependent enhancement of infection and IL-6 production

Jun Shimizu[1], Tadahiro Sasaki[2], Atsushi Yamanaka[1,3], Yoko Ichihara[1], Ritsuko Koketsu[2], Yoshihiro Samune[2], Pedro Cruz[1], Kei Sato[1], Naomi Tanga[1], Yuka Yoshimura[1], Ami Murakami[1], Misuzu Yamada[1], Kiyoe Itoi[1], Emi E. Nakayama[2], Kazuo Miyazaki[1,*], Tatsuo Shioda[2,3,*]

[1] MiCAN Technologies Inc., KKVP 1-36, Goryo-ohara, Nishikyo-Ku, Kyoto 615-8245, Japan

[2] Department of Viral Infection, Research Institute for Microbial Diseases, Osaka University, 3-1, Yamada-oka, Suita, Osaka 565-0871, Japan

[3] Mahidol-Osaka Center for Infectious Diseases, Faculty of Tropical Medicine, Mahidol University, Bangkok, Thailand

Supplemental Figure 1

Diagram of Mylc cell lines used in this study.

The immortalized myeloid cell lines (Mylc) were established from human iPS cells as mentioned in the section of Methods. The Mylc cell line (K-ML2 (AT)) was further cloned and selected based on their function (susceptibility for SARS-CoV-2 and productibility of IL-6). The resulting cell line, K-ML2 (AT) clone 35, was used as a host cell for SARS-CoV-2 infection in measuring ADE activity of sera. Another clone, clone 35-40, was used in measuring IL-6 production upon the stimulation with SARS-CoV-2 along with sera. The relationship of the parental cells and cloned cells are summarized.

Supplemental Figure 2

Infectibility of Mylc and Vero cells for dengue virus.

Five different and DC-differentiated Mylc lines (3×10^4/well, orange circles) and Vero cells (4×10^4/well, blue circles) were infected with the titrated amount of dengue virus type 2, strain 16681, in 96-well plates. After incubation at 37°C for 3 days, the SNs were harvested. Vero cells were cultured with the undiluted SNs, and cells were stained with 4G2 Ab (anti-Flavivirus Envelope protein Ab) three days later. The number of stained focuses per well was counted. More than 100 focuses are plotted as >100. The assays were performed in duplicate, and the result is expressed as an average.

Supplemental Figure 3

Changes in the expression of the CD14 cell surface marker.

K-ML2 and K-ML2 (AT) clone 35 cells before and after DC-differentiation were stained with FITC-conjugated anti-CD14 Ab in the presence of Fc-blocker (eBioscience). As a negative control, FITC-conjugated isotype-matched Ab was used.

Supplemental Figure 4

The expression of ACE2 and TMPRSS2 in Mylc cell lines.

K-ML2 and K-ML2 (AT) cells before and after DC-differentiation were analyzed for the expression of ACE2 and TMPRSS2 by qPCR. Error bars indicate SD. N=3.

Supplemental Figure 5

Microscopic observation of K-DC2 (AT) cells infected with SARS-CoV-2.

K-DC2 (AT) cells or K-DC2 (AT) clone 35 cells (2×10^4/well) were cultured in the presence (**b**, **d**) or absence (**a**, **c**) of SARS-CoV-2 (1,250 copies/µL). Three days later, cells were observed microscopically.

Supplemental Figure 6

Comparison of experimental protocols in SARS-CoV-2 infection.

(**a**) K-DC2, K-DC2 (AT), and no cells (indicated as None) were cultured with SARS-CoV-2 for 3 days without any washing. (**b**) Cells and viruses were mixed in tubes, and 4 h later, free viruses were washed out. Cells were cultured in 96-well plates for 3 days. (**c**) Cells and viruses were cultured in 96-well plates, and 4 h later, culture SNs containing free viruses were removed as much as possible. Fresh medium was added, and cells were cultured for 3 days. The amounts of viruses in SNs after 3 days of culture were measured by qPCR (top panels), and fold increase was calculated as in Fig. 1 (bottom panels). Error bars indicate SD. N=3 (**a**, **b**), N=2 (**c**).

Supplemental Figure 7

K-ML2 cells express functional Fc-receptor.

K-ML2 cells were stained with the Ab (PE-anti-human TCR Vβ8 Ab, mouse IgG_{2a}, BioLegend) in the presence or absence of Fc-blocker (eBioscience). As a negative control, unstained cells were used.

Supplemental Figure 8

Infection-enhancing activity of serum from mice immunized with SARS-CoV-2.

K-ML2 (AT) clone 35 cells (2×10^4/well) were cultured with a constant amount of SARS-CoV-2 (1,250 copies/μL) in the presence or absence (control culture) of serially-diluted serum from (**a**) a normal mouse or (**b**) mice immunized with SARS-CoV-2. Three days later, the culture SNs were harvested, and the amount of virus was determined by qPCR (N=3). The increment of viruses is expressed as the fold increase compared with the amount of viruses in control culture (N=6). Red or black lines on the y-axis indicate the mean of control culture + a three or six SD cut-off, respectively. The black dotted line indicates fold increase = 1. (**c**) The region indicated with a dotted line in (**b**) is shown on a log-log scale.

Supplemental Figure 9

Infection-enhancing activity of serum derived from patients infected with SARS-CoV-2.

As shown in the Fig. 3 legend, the results from ADE assays were classified into (**a**) Apparent ADE, (**b**) Slight ADE, and (**c**) no ADE (None) groups. The results from all patients (N=100) are shown in Supplemental Fig. 9 and Fig. 3**a**.

Supplemental Figure 10

ADE observed by sera derived from COVID-19 patients depends on FcR.

(Left panel) 4G2 Ab was used as FcR-binding competitor. As shown in left panel, the presence of 4G2 could inhibit the staining of K-ML2 (AT) clone 35 cells with anti-hTCR Ab (blue histogram), demonstrating that the staining with the irrelevant anti-hTCR Ab (red) is mediated by FcR and that 4G2 can also bind to FcR. Monoclonal antibody 4G2 (mouse IgG_{2a}, flavivirus group cross-reactive) was purchased from American Type Culture Collection (Manassas, VA). (Right panel) K-ML2 (AT) clone 35 cells were cultured with a constant dose of patient's serum and SARS-CoV-2 in the presence or absence of the titrated amount of 4G2 Ab. N=3. Serum from patient #96, #8 and #58 represents ADE-causing serum. Three days later, the amount of viruses in culture supernatants was measured by qPCR. The amount of viruses in the absence of 4G2 or 4G2 and patient's serum was regarded as 100% or 0% ADE response, respectively.

Supplemental Figure 11

K-ML2 (AT) cells can also produce IL-6 only in the presence of serum derived from COVID-19 patient.

K-ML2 (AT) clone 35 cells (top panels) or re-cloned 35-40 cells (bottom panels) were cultured with different doses of SARS-CoV-2 as indicated in the presence (blue circles) or absence (orange circles) of serially-titrated serum from COVID-19 patient #73. Orange circles indicate the culture of cells alone. Three days later, the amount of IL-6 in SNs was measured and is shown as OD value. Error bars indicate SD. N=3.

Supplemental Figure 12

IL-6 production-enhancing activity of sera derived from SARS-CoV-2-sensitized hosts.

(**a**) K-ML2 (AT) clone 35-3, 35-20, or 35-40 cells. (**b**) Clone 35-40 cells were cultured with the titrated amount of SARS-CoV-2 in the presence or absence of a constant dose (final ×100 dilution) of serum from COVID-19 patients or healthy control donors (HC) as indicated. Patient-derived sera #32, #38, and #99 belong to the A-2 subgroup in ADE assay, and serum #62 belongs to the No ADE group. (**c**) Clone 35-40 cells were cultured with SARS-CoV-2 (1×10^4 copies/μL) and serially-titrated serum from SARS-CoV-2-immunized or normal mice. The amount of IL-6 in each SN after three days' culture was measured. The amount of IL-6 is plotted as the OD value in the ELISA assay. Error bars indicate SD. N=3.

Supplemental Figure 13

IL-6 production-enhancing activity of sera derived from patients infected with SARS-CoV-2.

K-ML2 (AT) clone 35-40 cells (2×10^4/well in a 96-well flat plate) were cultured with (blue circles) or without (orange circles) SARS-CoV-2 (1×10^4 copies/μL) in the presence or absence

of serially-diluted serum from COVID-19 patients (N=3). Three days later, the amount of IL-6 in culture SNs was measured by ELISA. Error bars indicate SD. The position of each result corresponds to that in Fig. 3a and Supplemental Fig. 9.

Supplemental Figure 14
Enhanced IL-6 production observed by sera derived from COVID-19 patients depends on FcR.
K-ML2 (AT) clone 35-40 cells were cultured with a constant dose of patient's serum and SARS-CoV-2 in the presence or absence of the titrated amount of 4G2 Ab. N=3. Serum from patient #24, #73 and #78 represents IL-6 production-enhancing serum. 4G2 Ab was used as FcR-binding competitor reagent. Three days later, the amount of IL-6 in culture SNs was measured by IL-6 ELISA. The amount of IL-6 in the absence of 4G2 or 4G2 and patient's serum was regarded as 100% or 0% IL-6 production, respectively. The percent inhibition of IL-6 production by 4G2 Ab is plotted in y-axis.

Supplemental Table 1
Information about COVID-19 patients (sex and age).

Supplemental Table 2
The amount of anti-SARS-CoV-2 IgG in each serum sample from COVID-19 patients.
The amount of anti-SARS-CoV-2 IgG in each serum sample from COVID-19 patients was measured using VITROS™ Anti-SARS-CoV-2 Total Reagent Pack (data given by REPROCELL (Kanagawa, Japan)).

Supplemental Table 3
Raw data of control cultures in qRT-PCR experiments.
In one qRT-PCR experiment to measure the amount of SARS-CoV-2, the control cultures (cells plus SARS-CoV-2 without serum) were set in 6–9 wells. This Table summarizes the sample names examined, the raw data (mean and SD) of the control cultures, and the value of fold increase calculated from the mean and SD in each experiment.

Supplemental Fig. 1

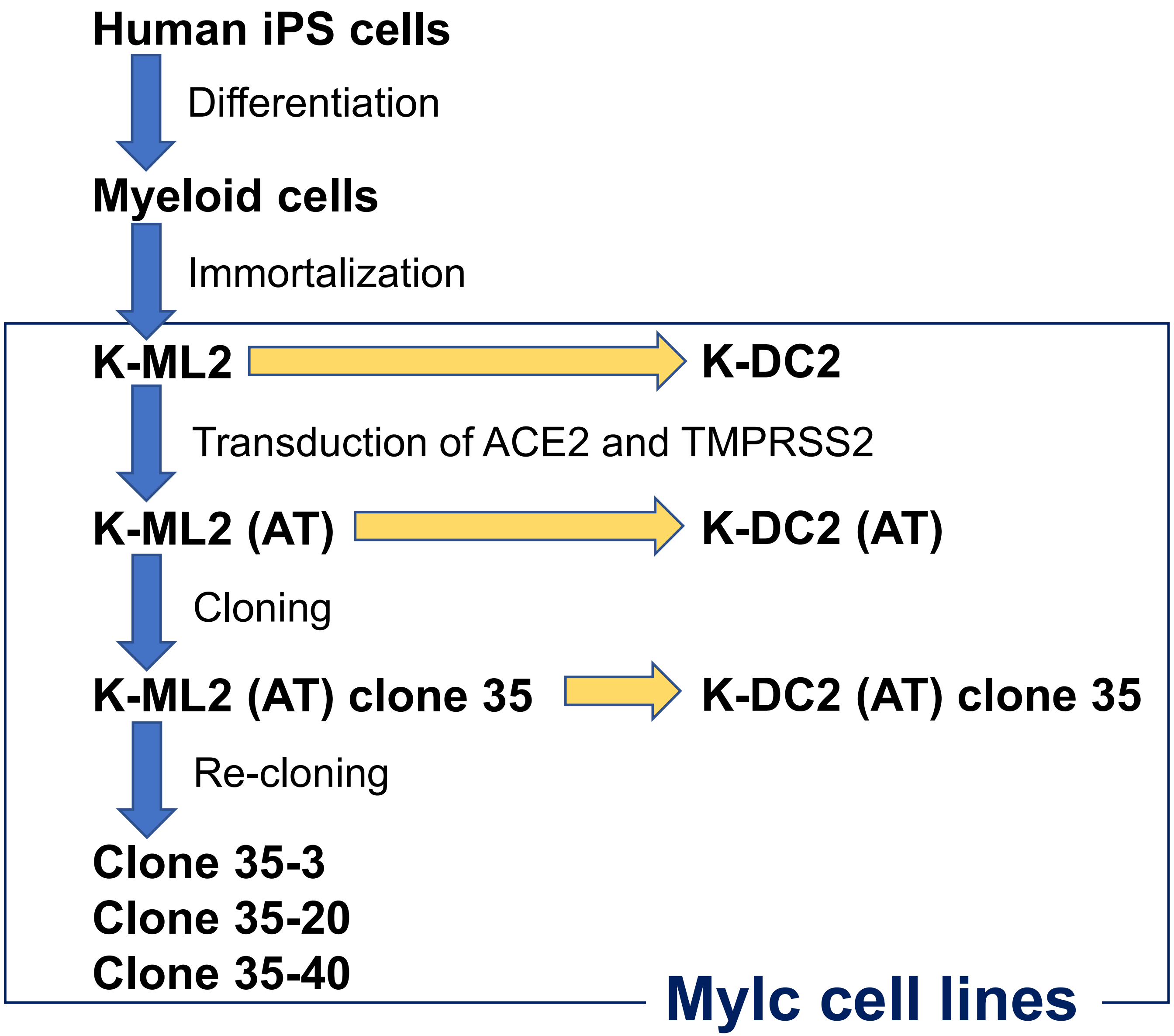

Supplemental Fig. 2

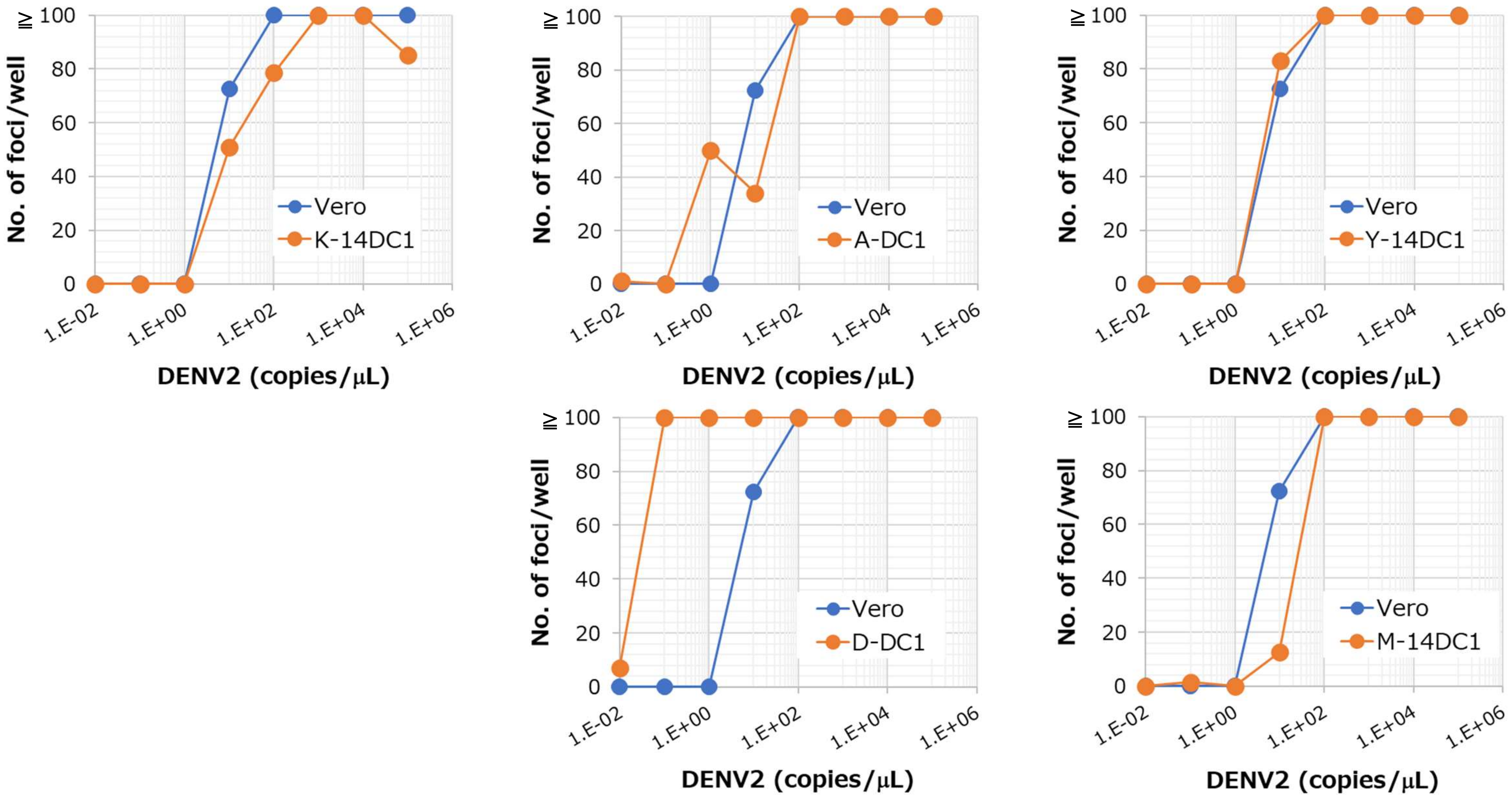

Supplemental Fig. 3

a

Group	Cells	Staining
	K-DC2	anti-CD14 Ab
		isotype control
	K-ML2	anti-CD14 Ab
		isotype control

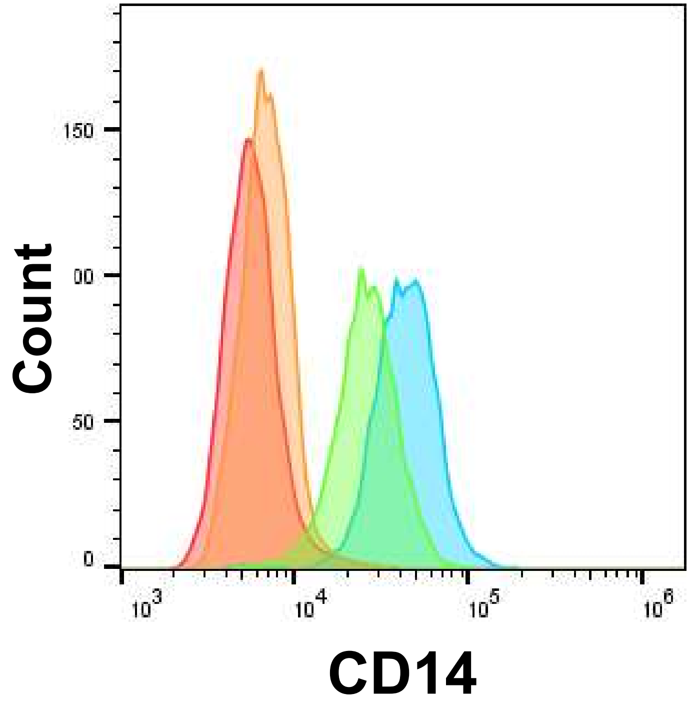

b

Group	Cells	Staining
	K-DC2(AT) clone 35	anti-CD14 Ab
		isotype control
	K-ML2(AT) clone 35	anti-CD14 Ab
		isotype control

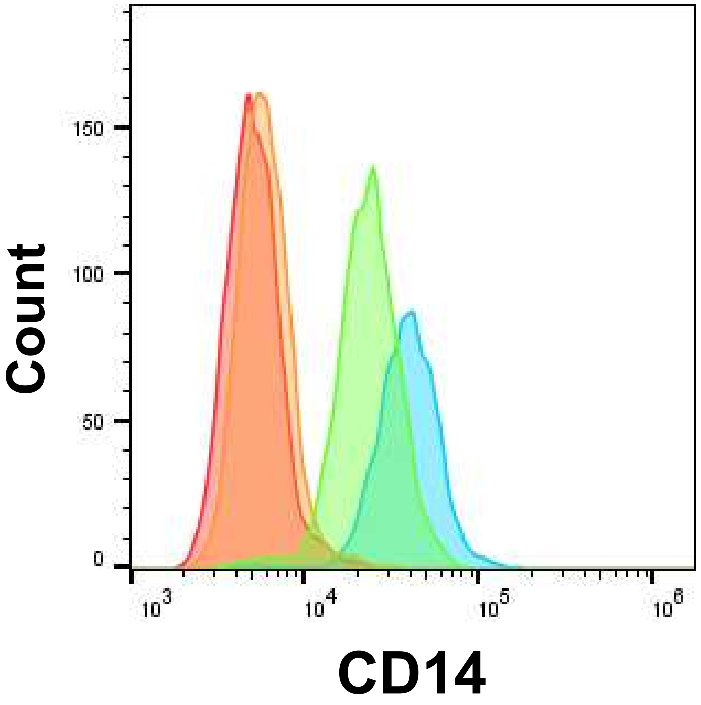

Supplemental Fig. 4

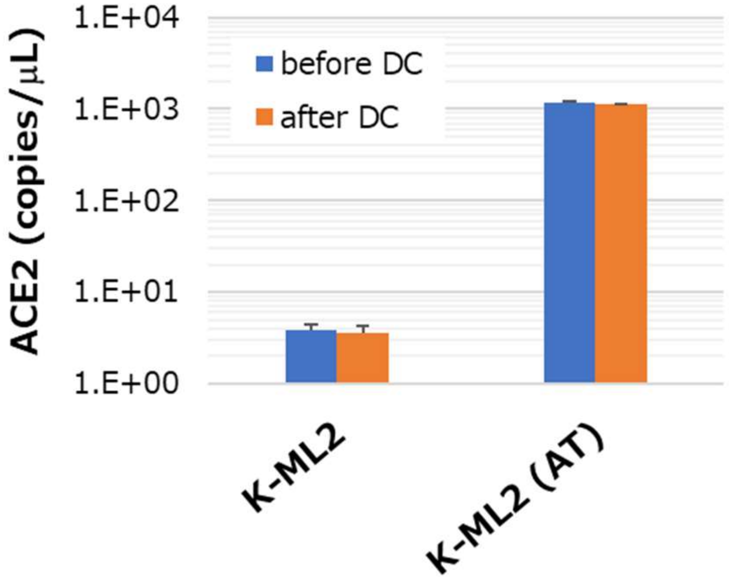

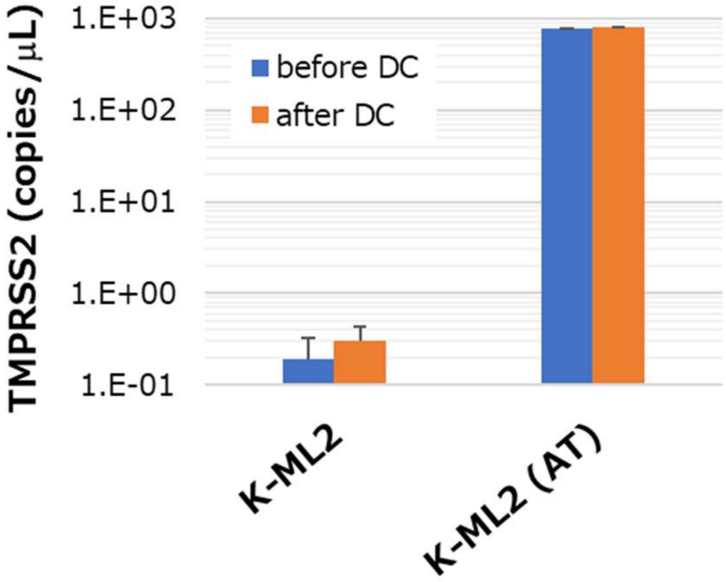

Supplemental Fig. 5

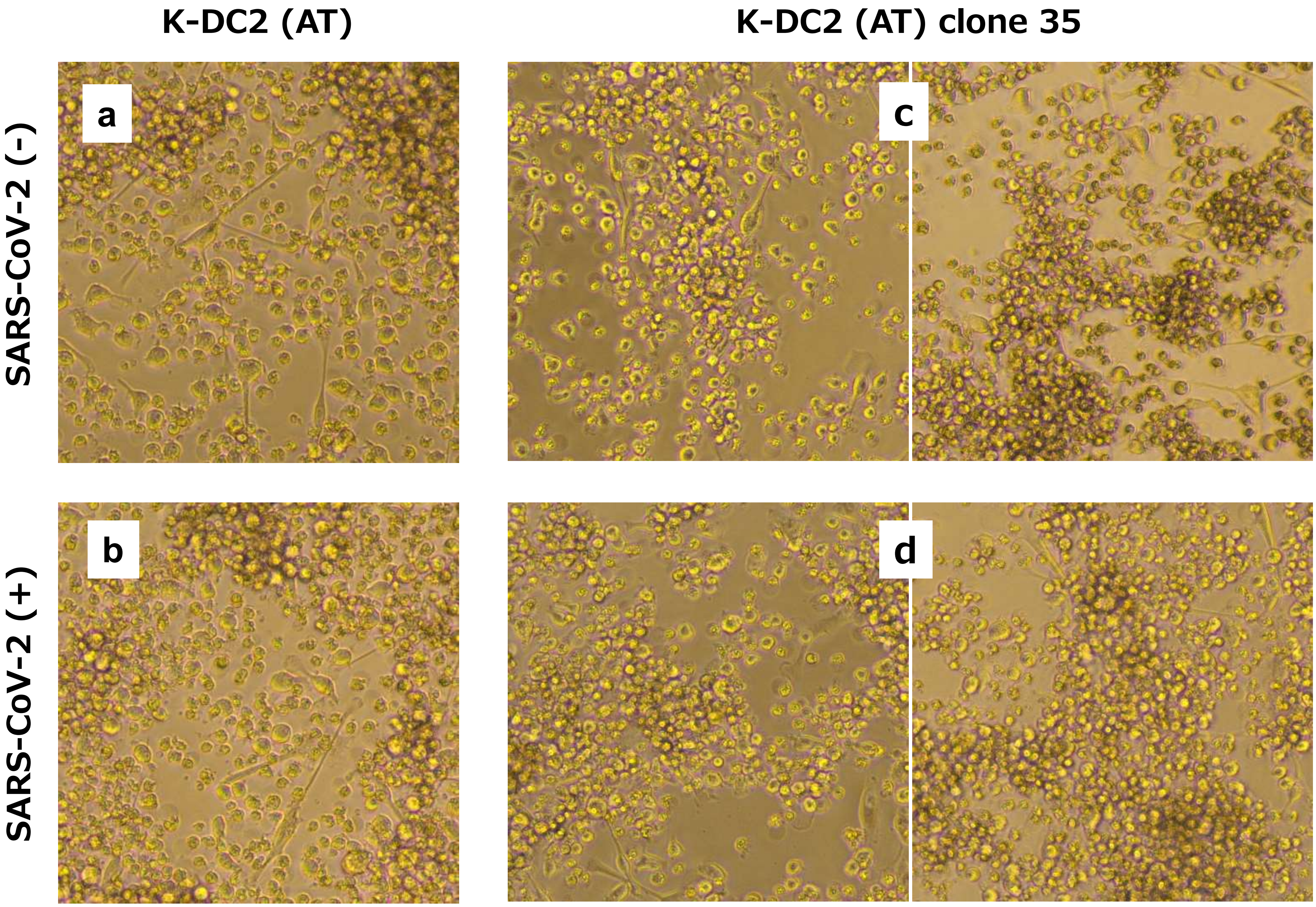

Supplemental Fig. 6

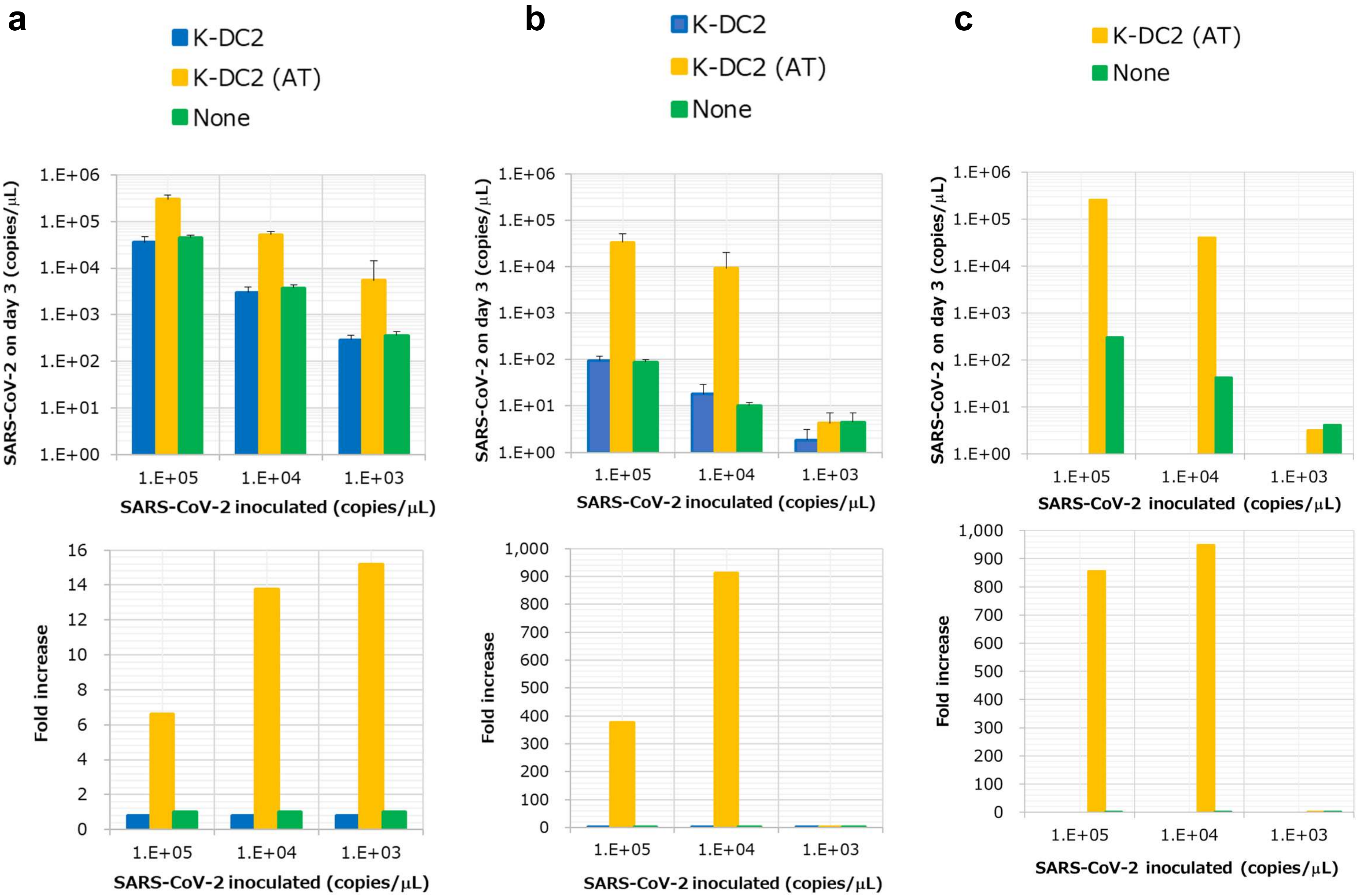

Supplemental Fig. 7

Group	Cells	Staining
	K-ML2	none
		anti-hTCR Vβ8
		Fc block ➡ anti-hTCR Vβ8

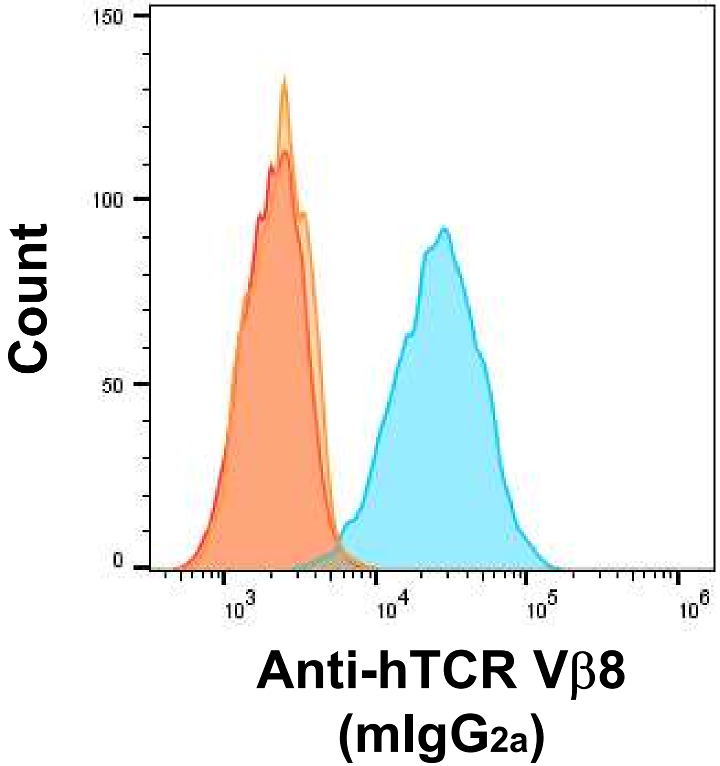

Supplemental Fig. 8

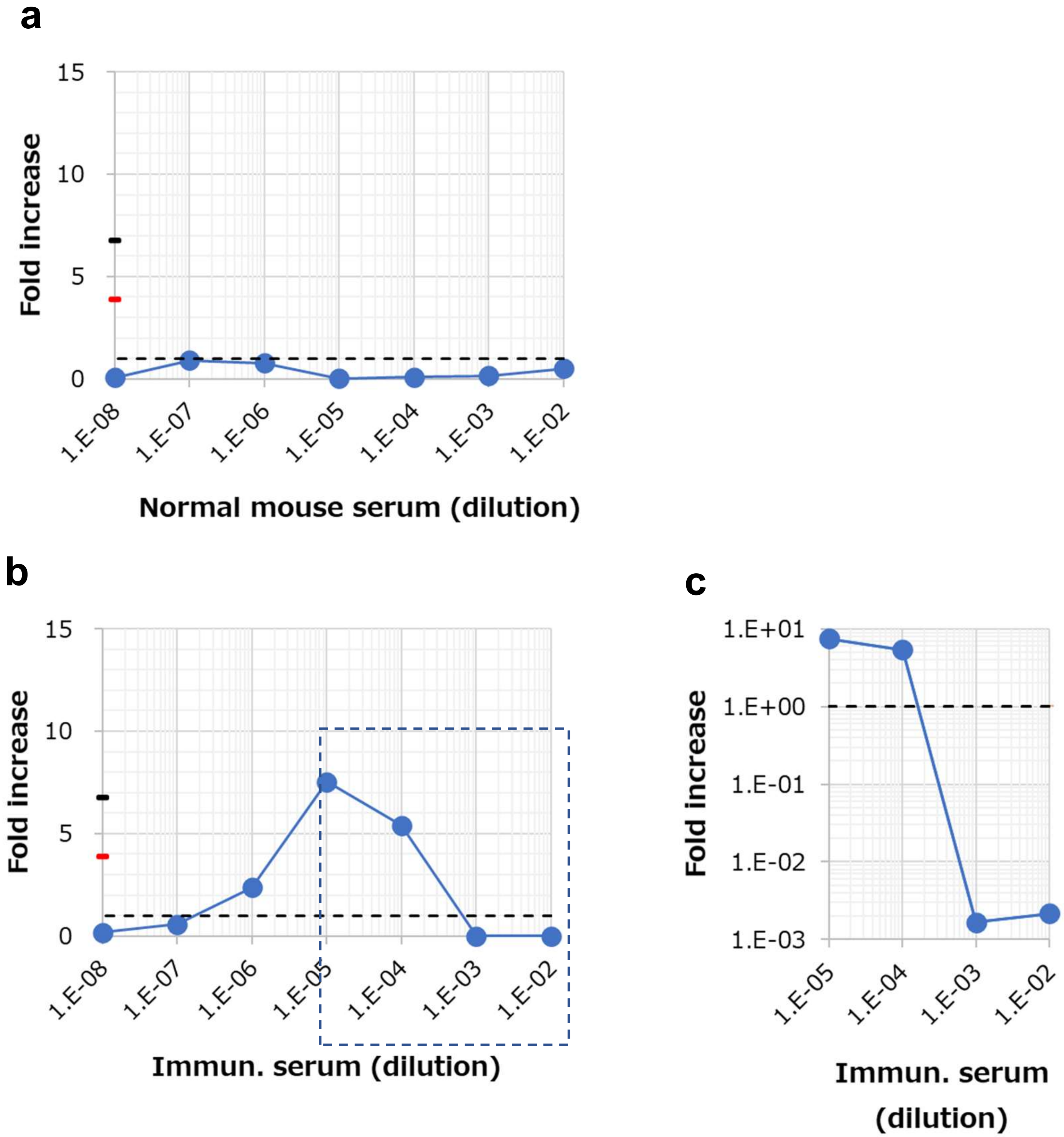

Supplemental Fig. 9-1

(a) Apparent ADE (A-1)

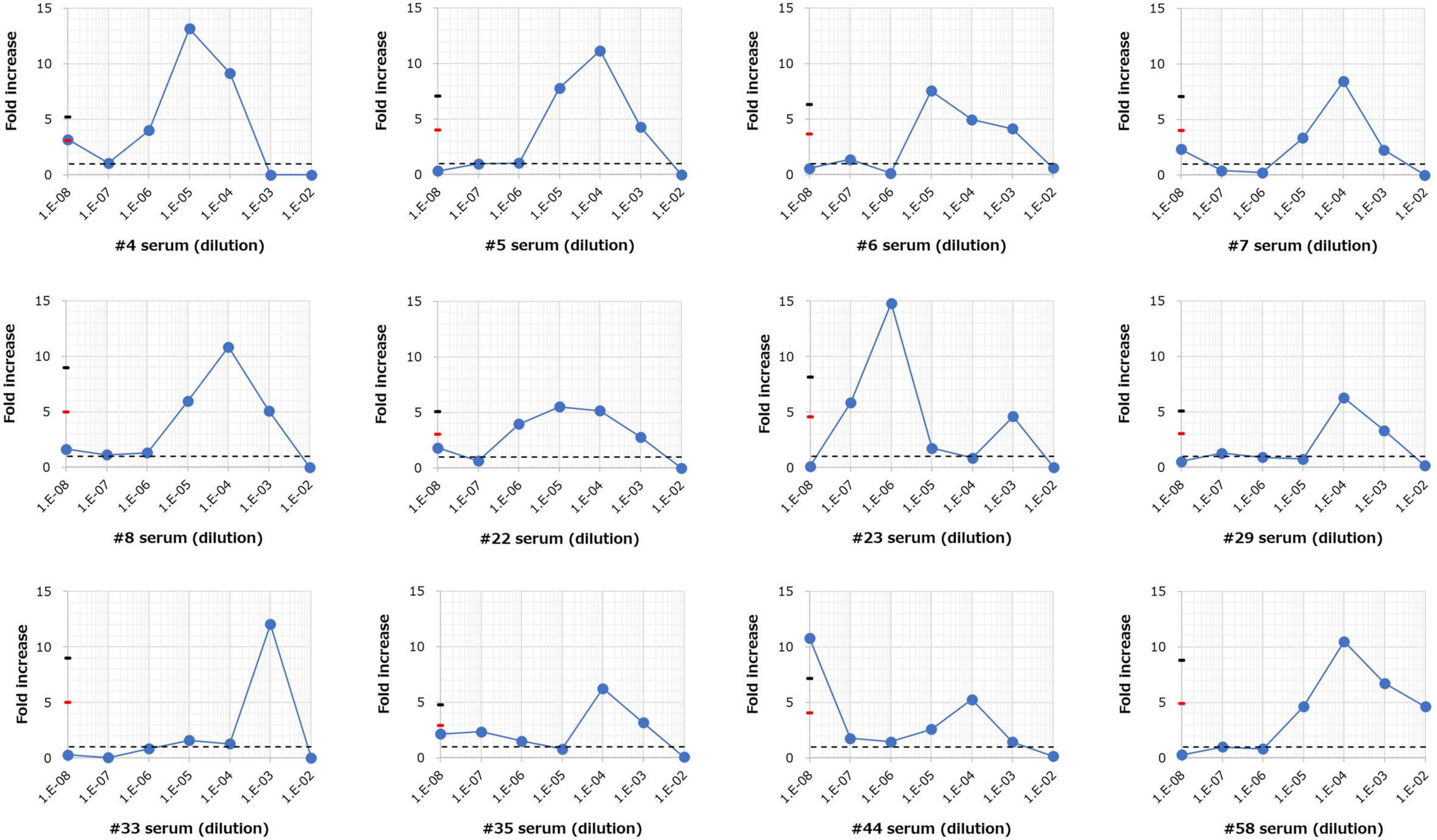

Supplemental Fig. 9-2

(a) Apparent ADE (A-1)

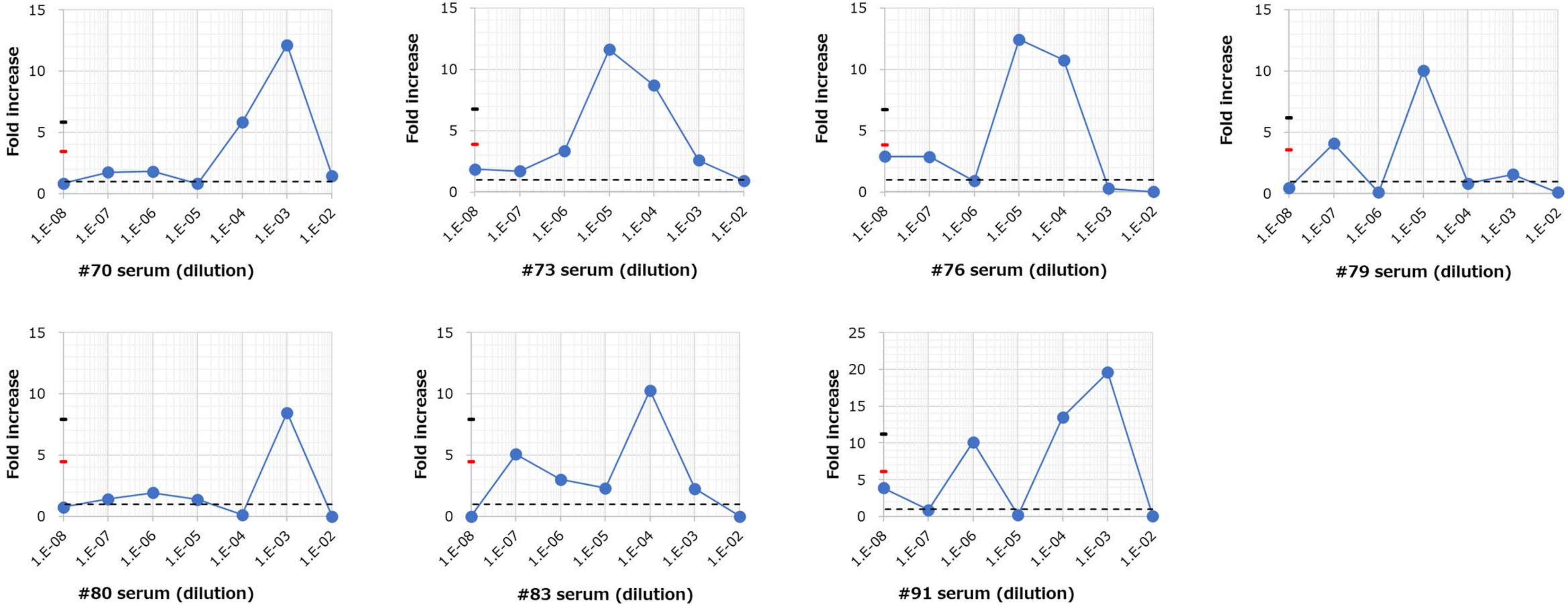

Supplemental Fig. 9-3

(a) Apparent ADE (A-2)

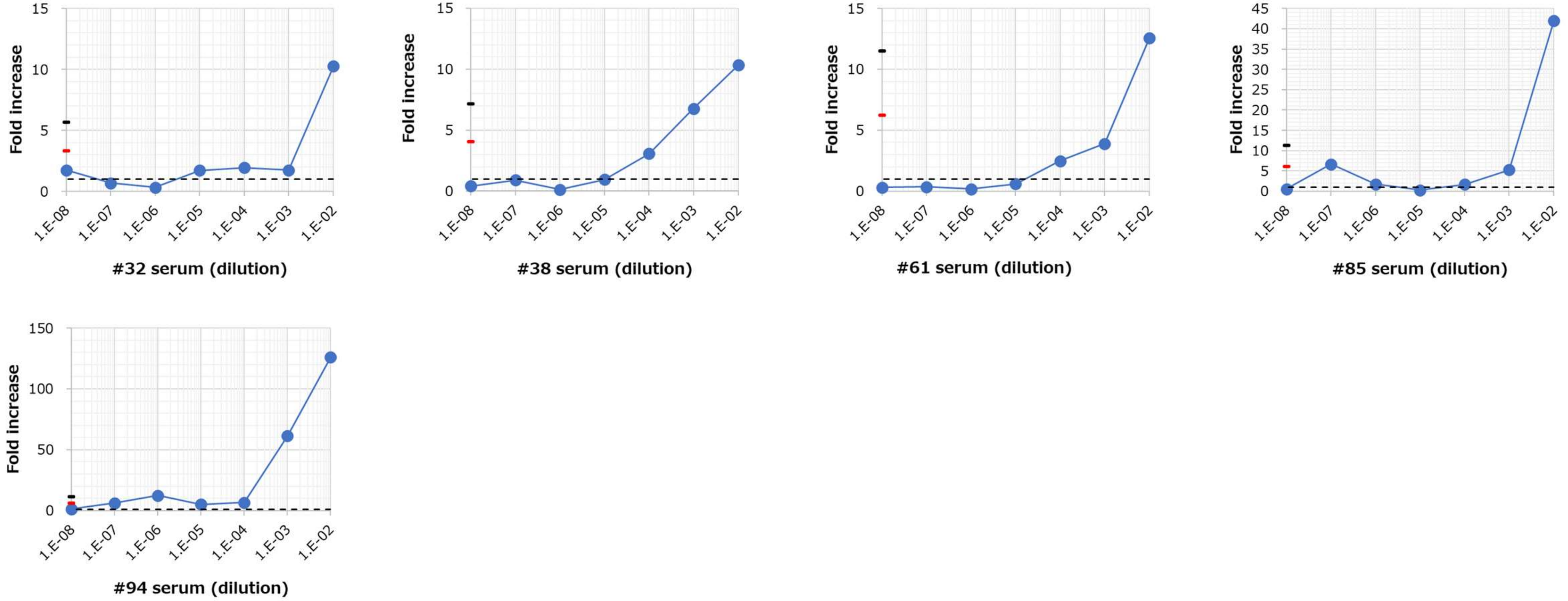

Supplemental Fig. 9-4

(b) Slight ADE

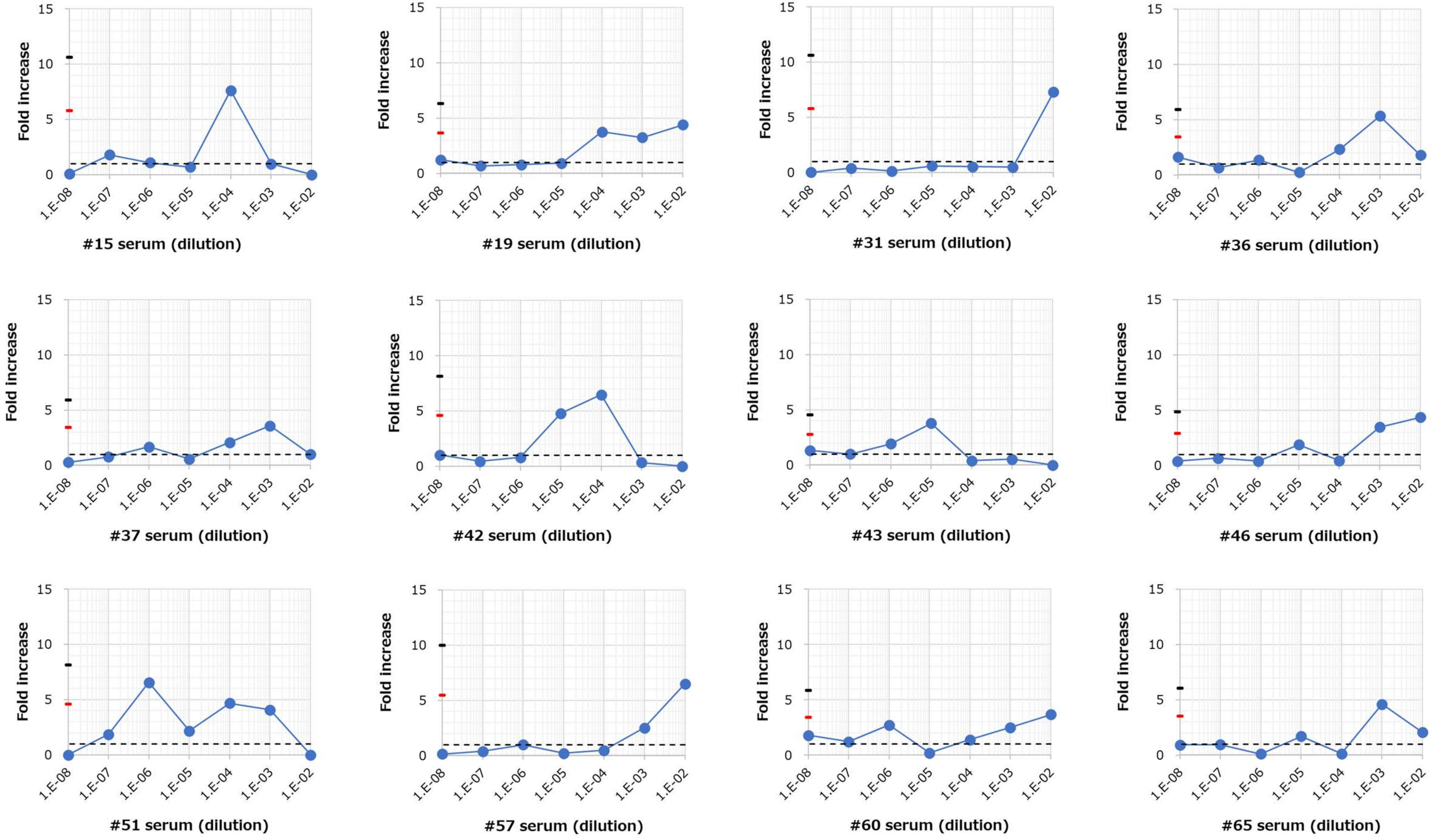

Supplemental Fig. 9-5

(b) Slight ADE

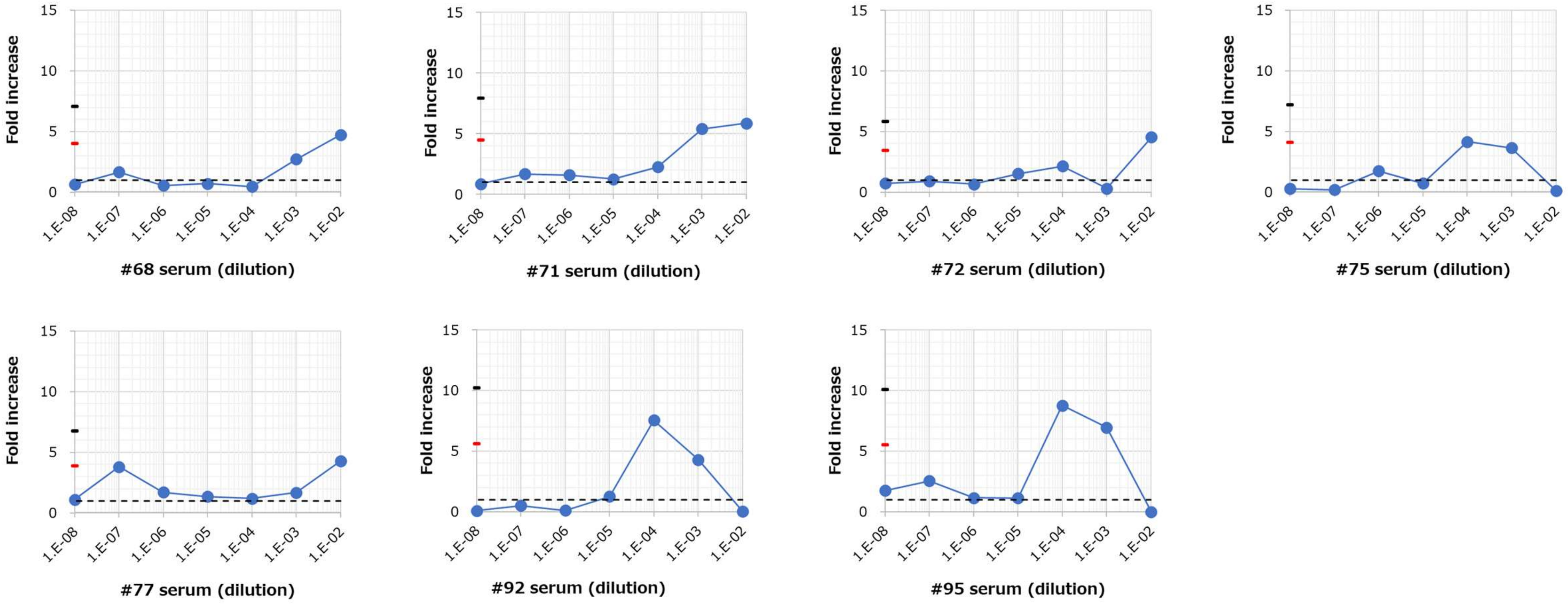

Supplemental Fig. 9-6

(c) No ADE

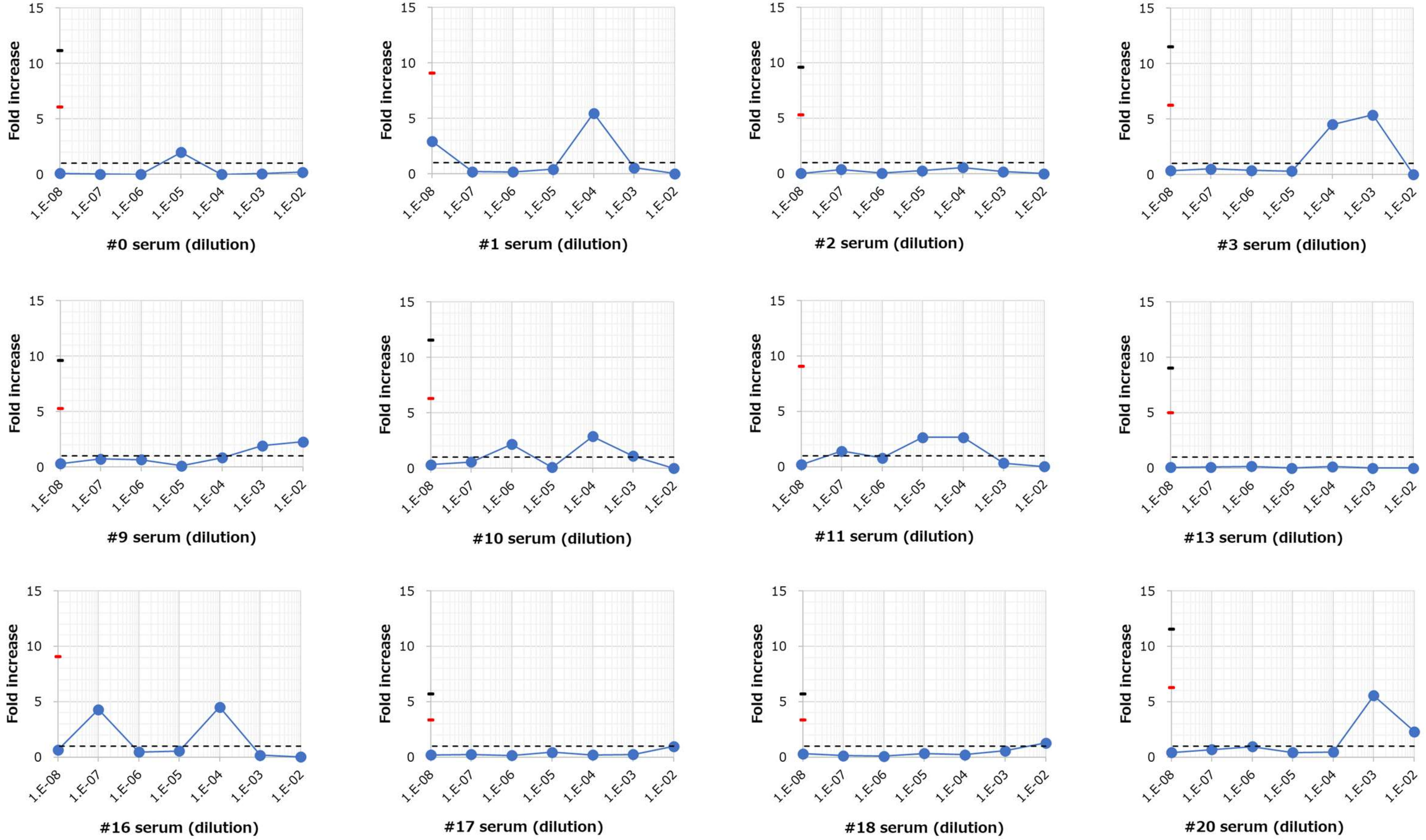

Supplemental Fig. 9-7

(c) No ADE

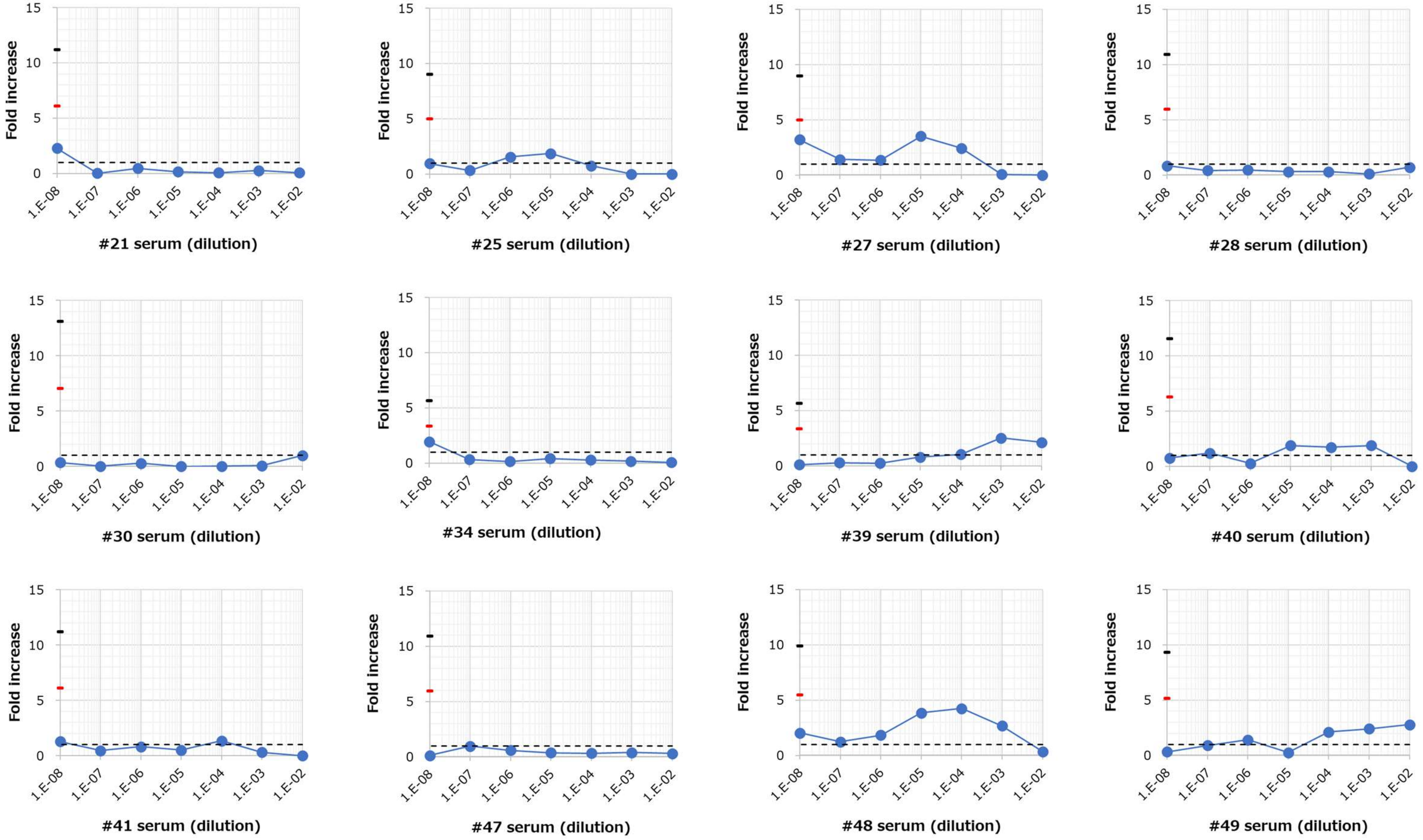

Supplemental Fig. 9-8

(c) No ADE

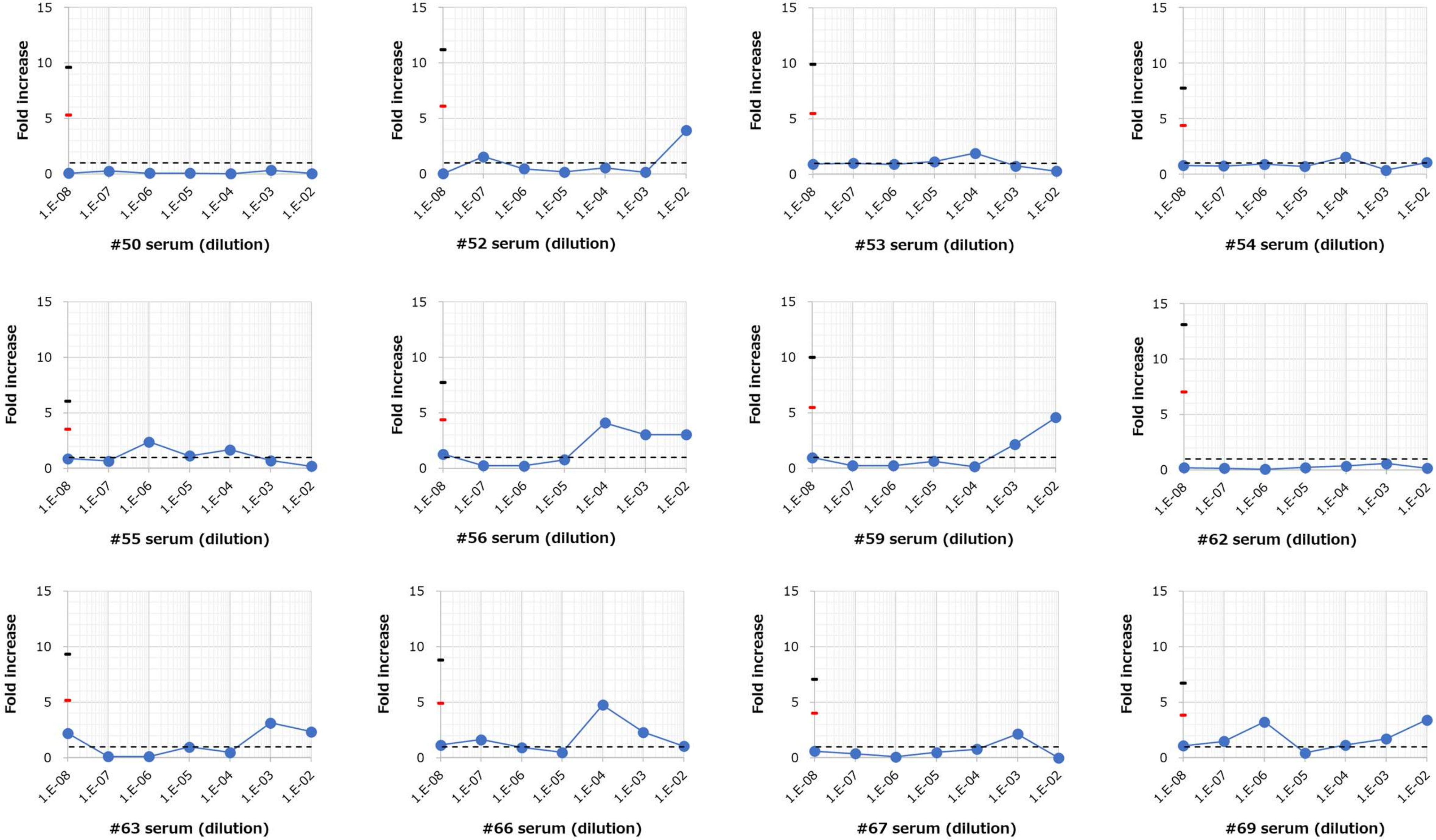

Supplemental Fig. 9-9

(c) No ADE

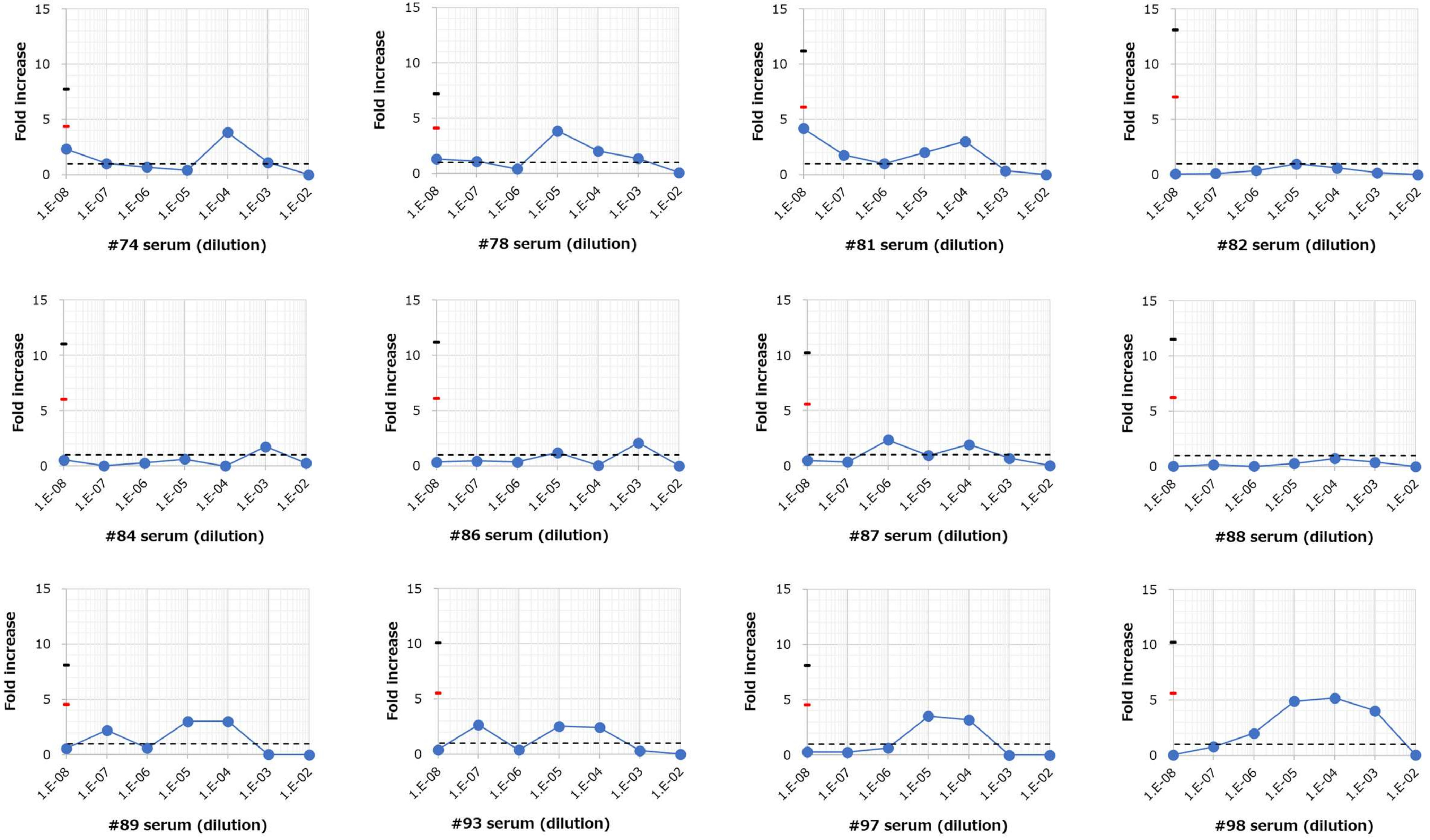

Supplemental Fig. 10

Group	Cells	Staining
	K-ML2 (AT) clone 35	none
		anti-hTCR Vβ8
		4G2 ➡ anti-hTCR Vβ8

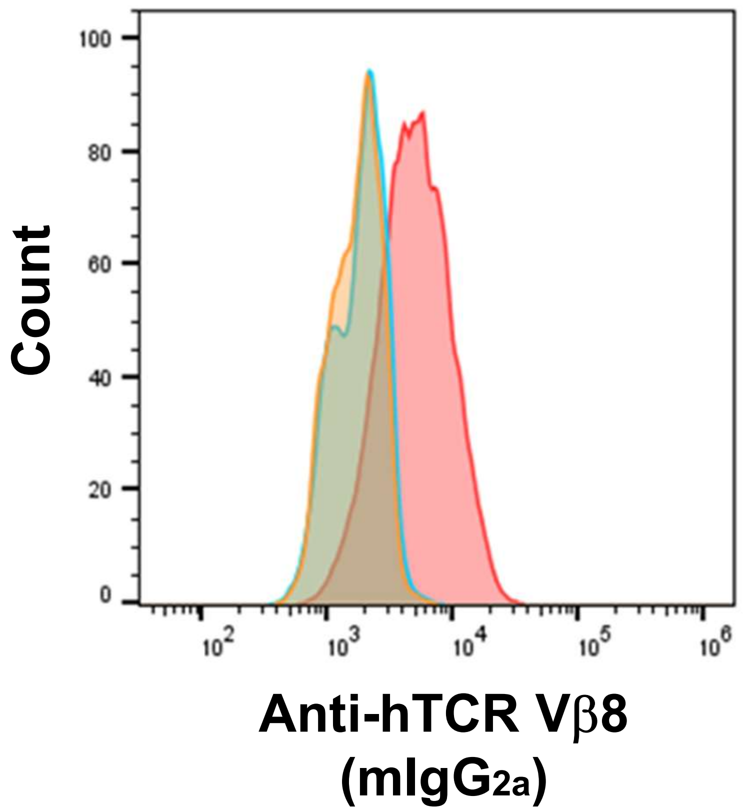

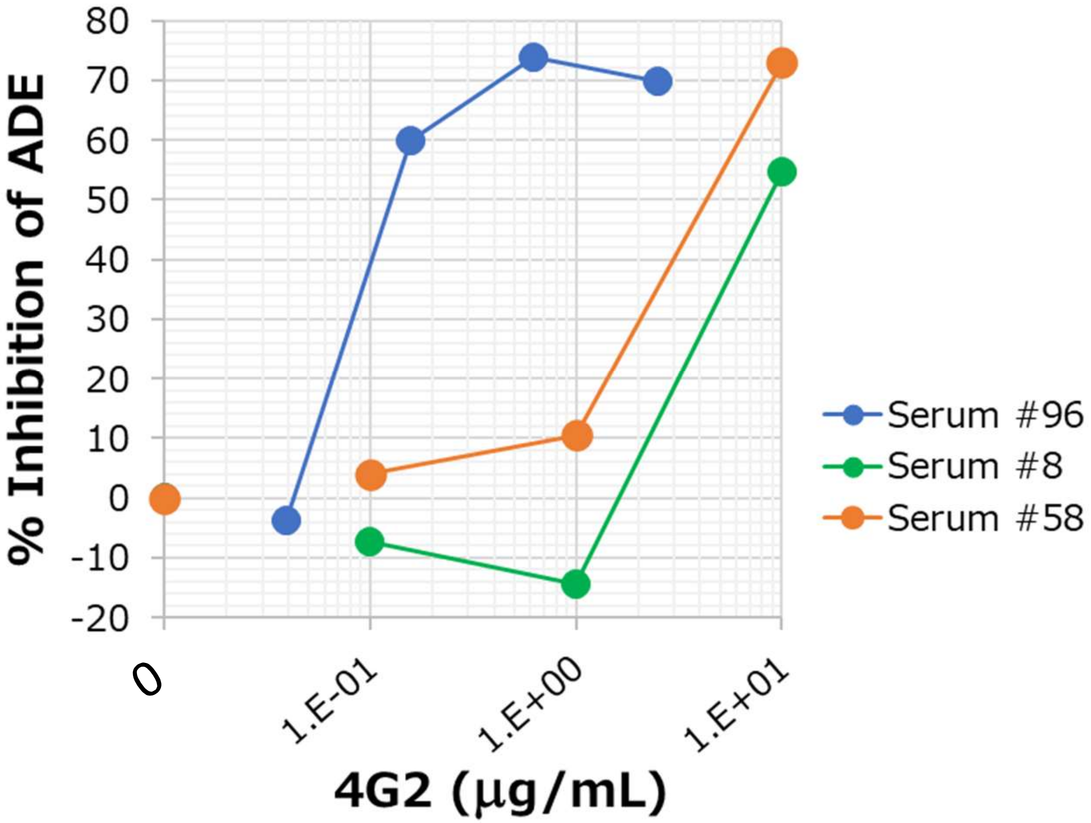

Supplemental Fig. 11

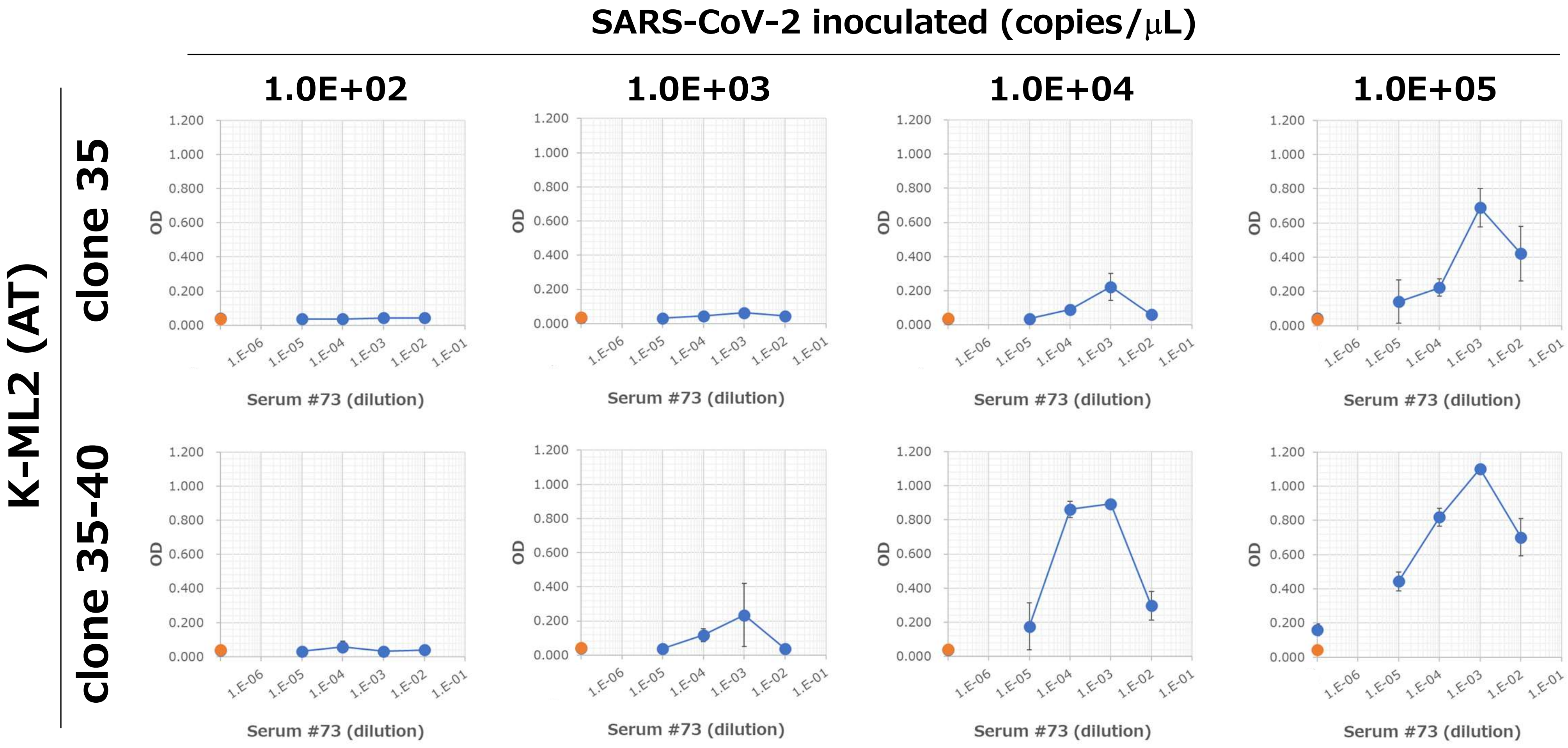

Supplemental Fig. 12

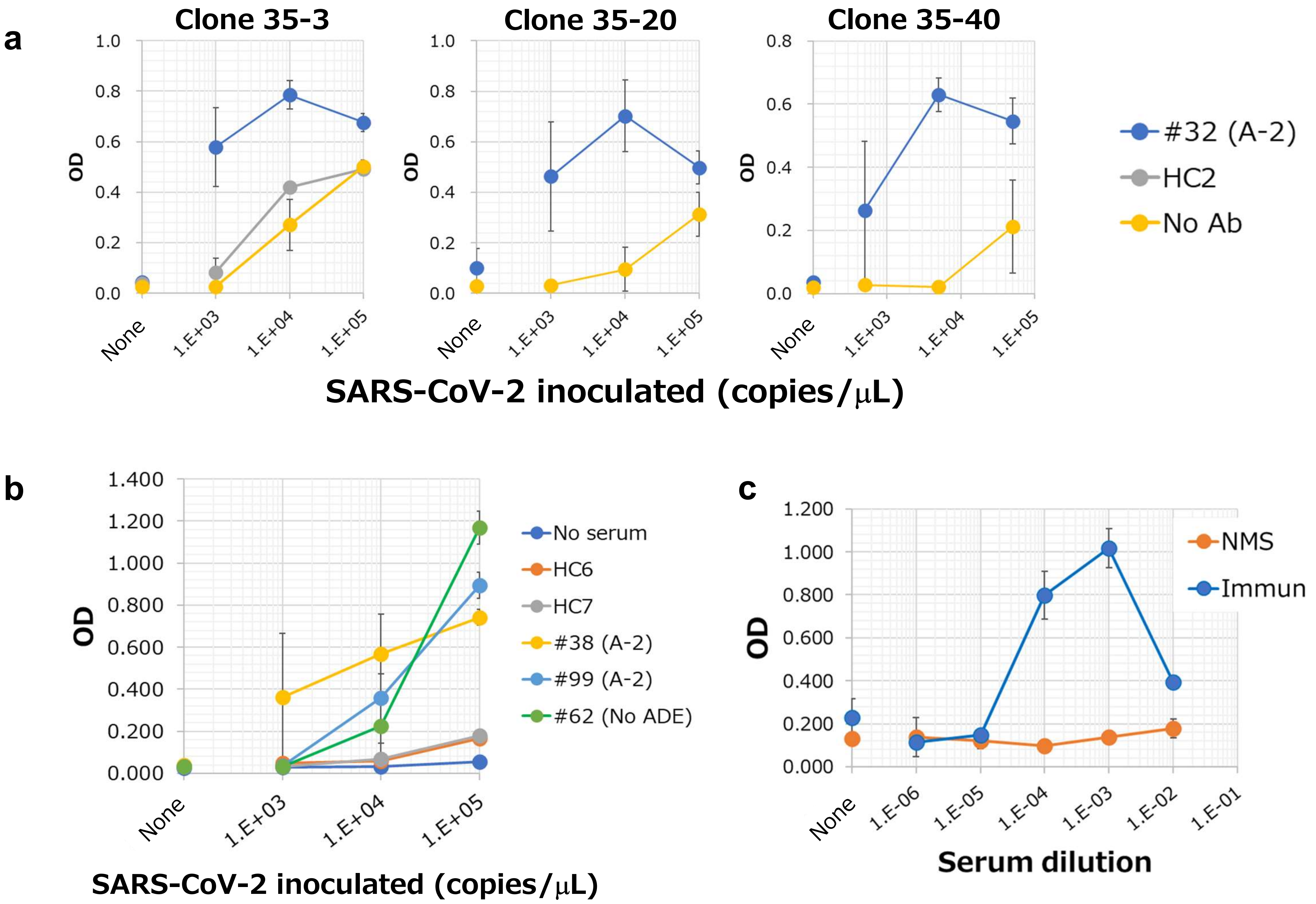

Supplemental Fig. 13

Severe COVID-19

ADE

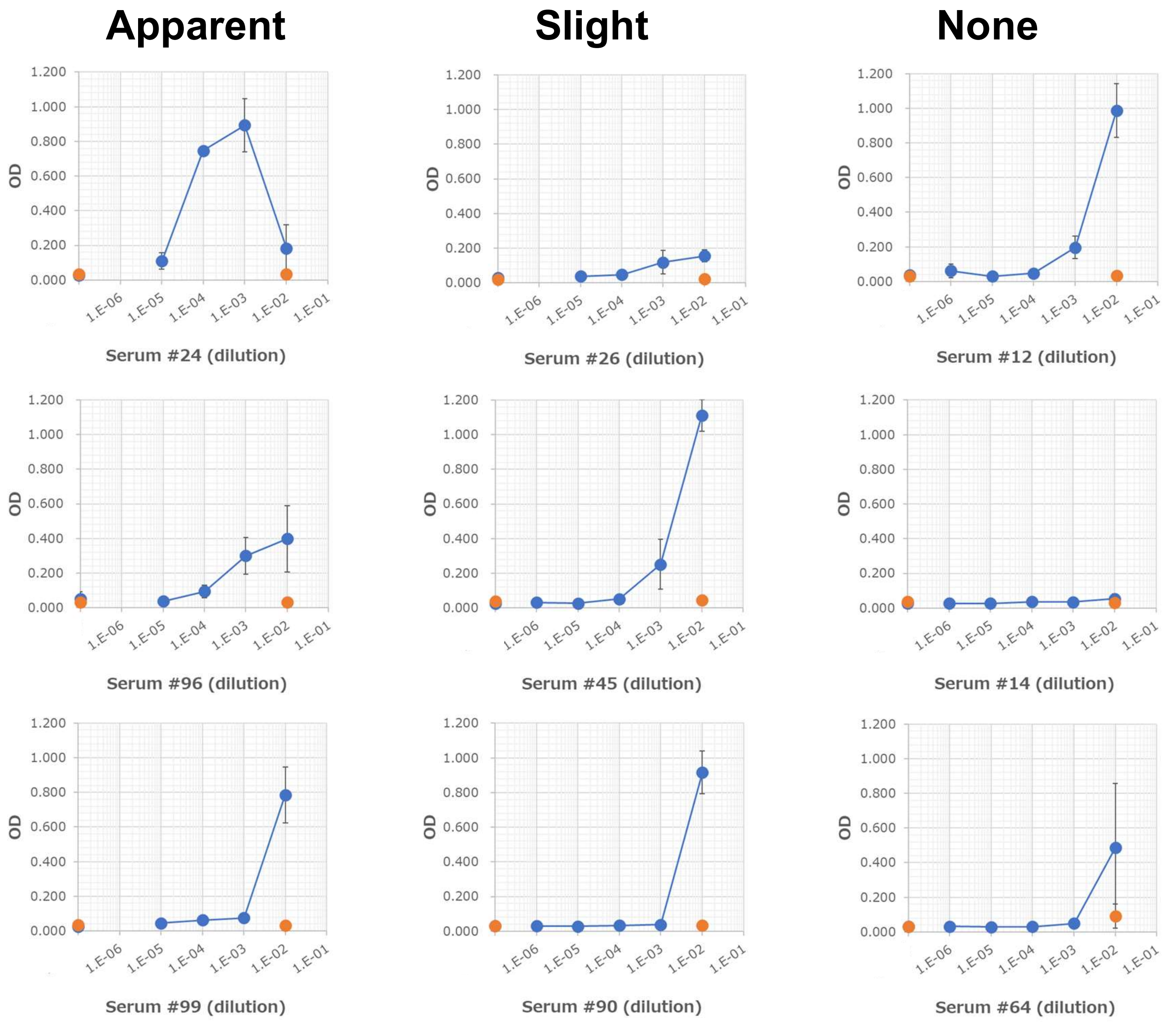

Supplemental Fig. 13-1

(a) Apparent ADE (A-1)

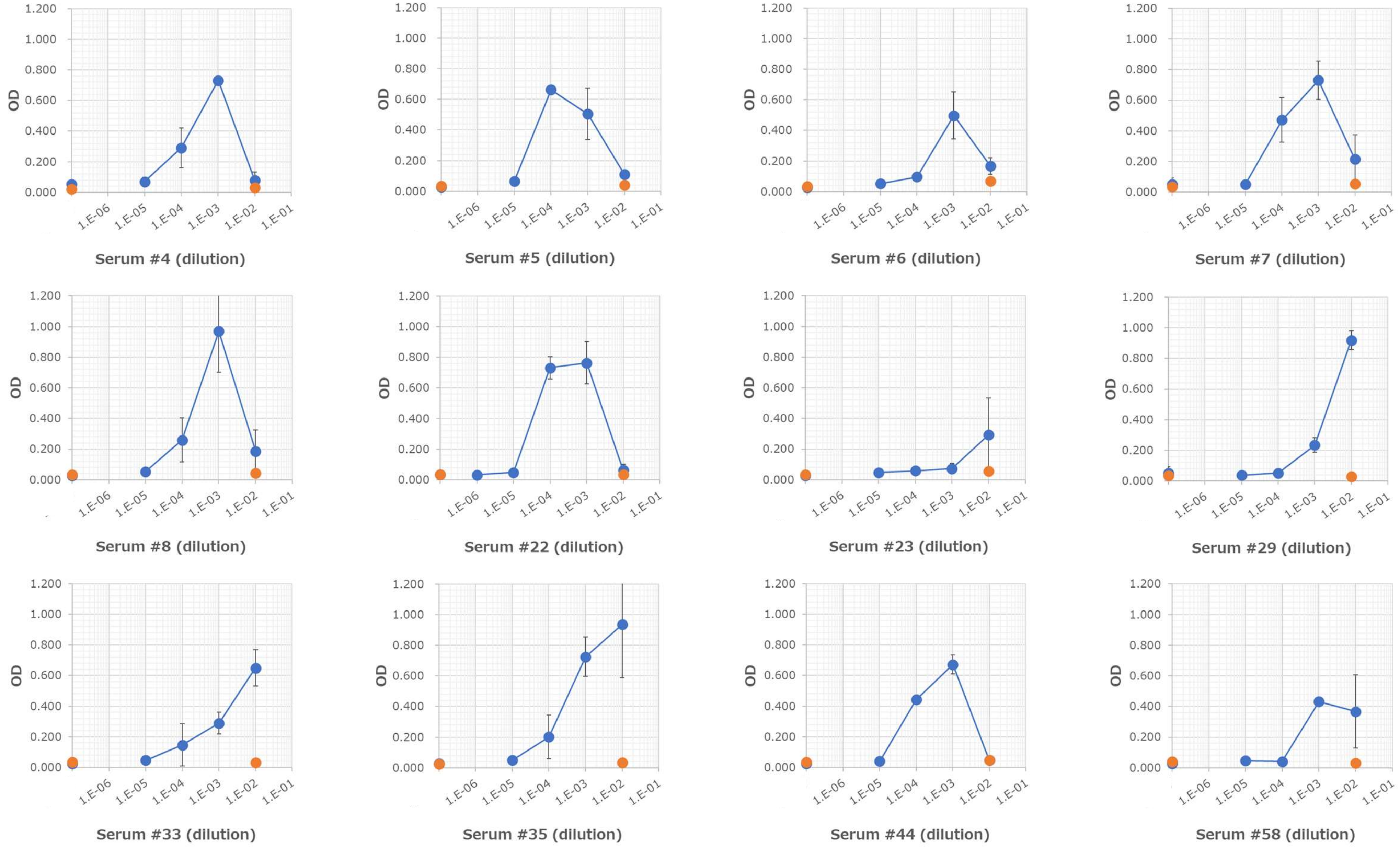

Supplemental Fig. 13-2

(a) Apparent ADE (A-1)

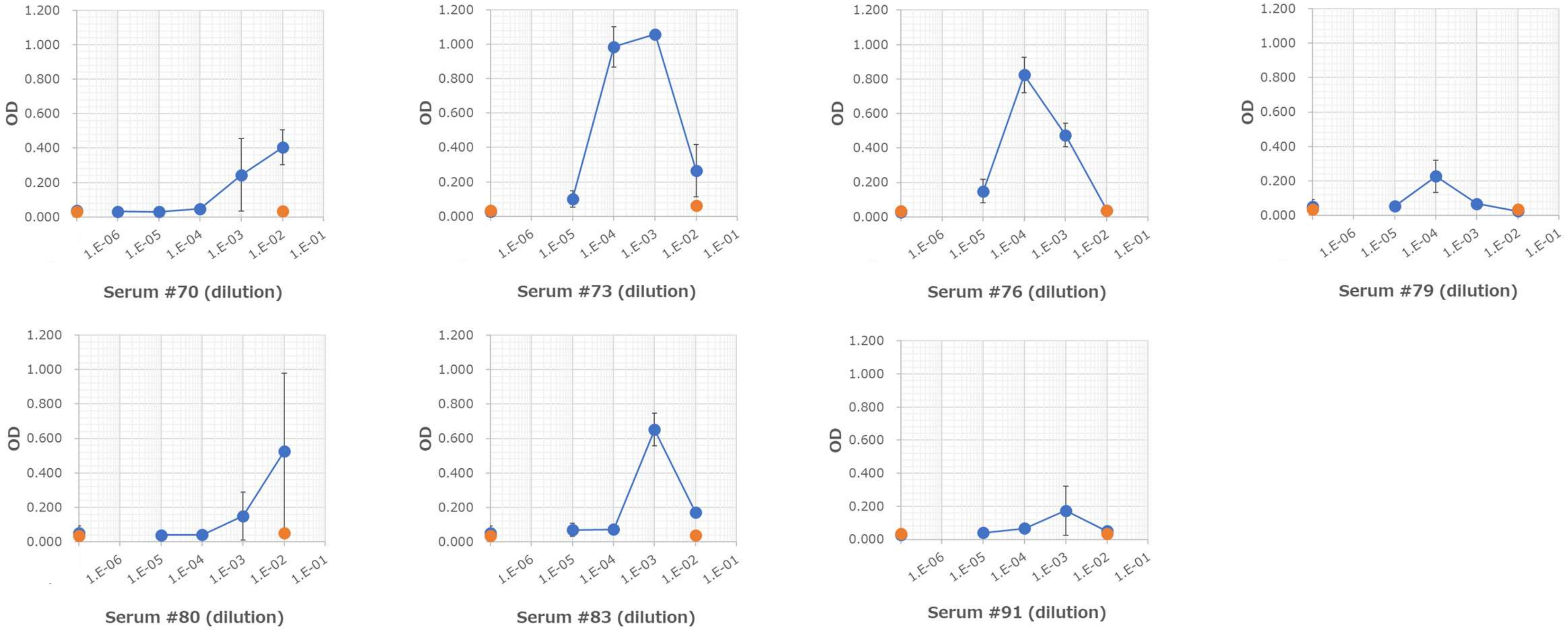

Supplemental Fig. 13-3

(a) Apparent ADE (A-2)

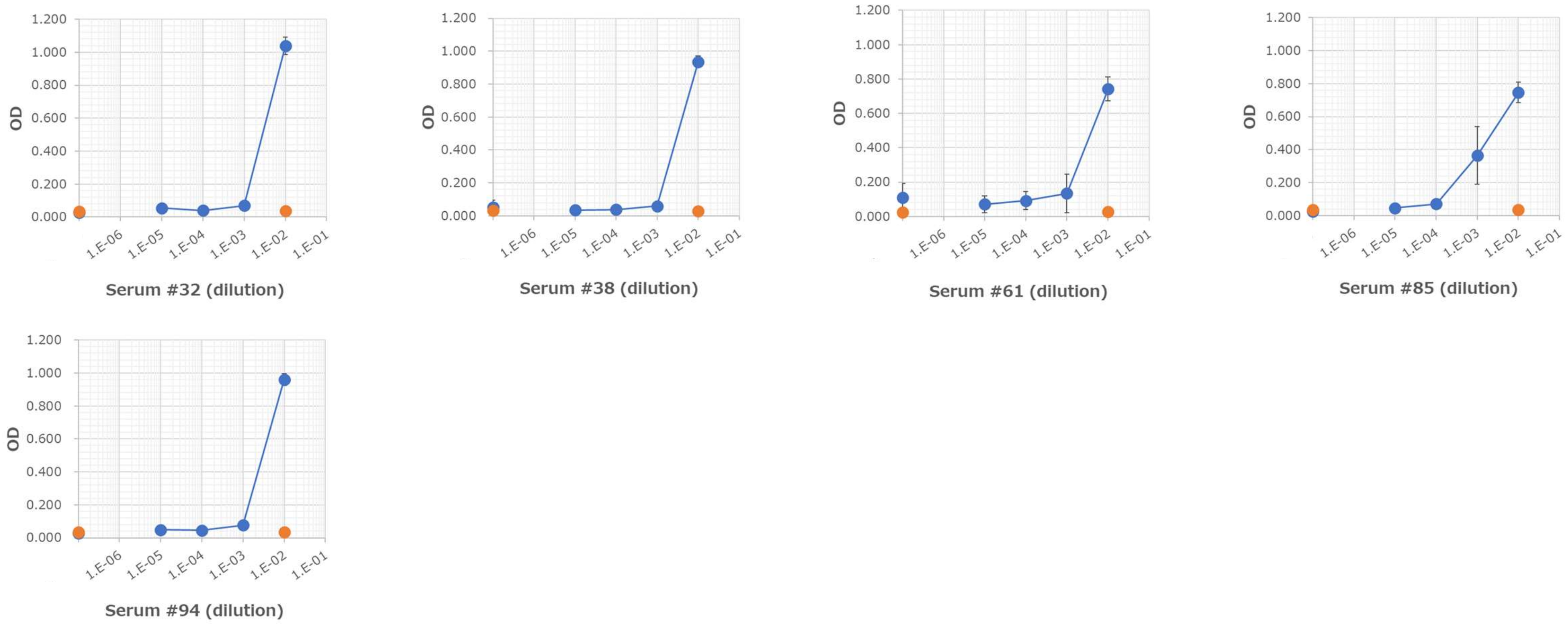

Supplemental Fig. 13-4

(b) Slight ADE

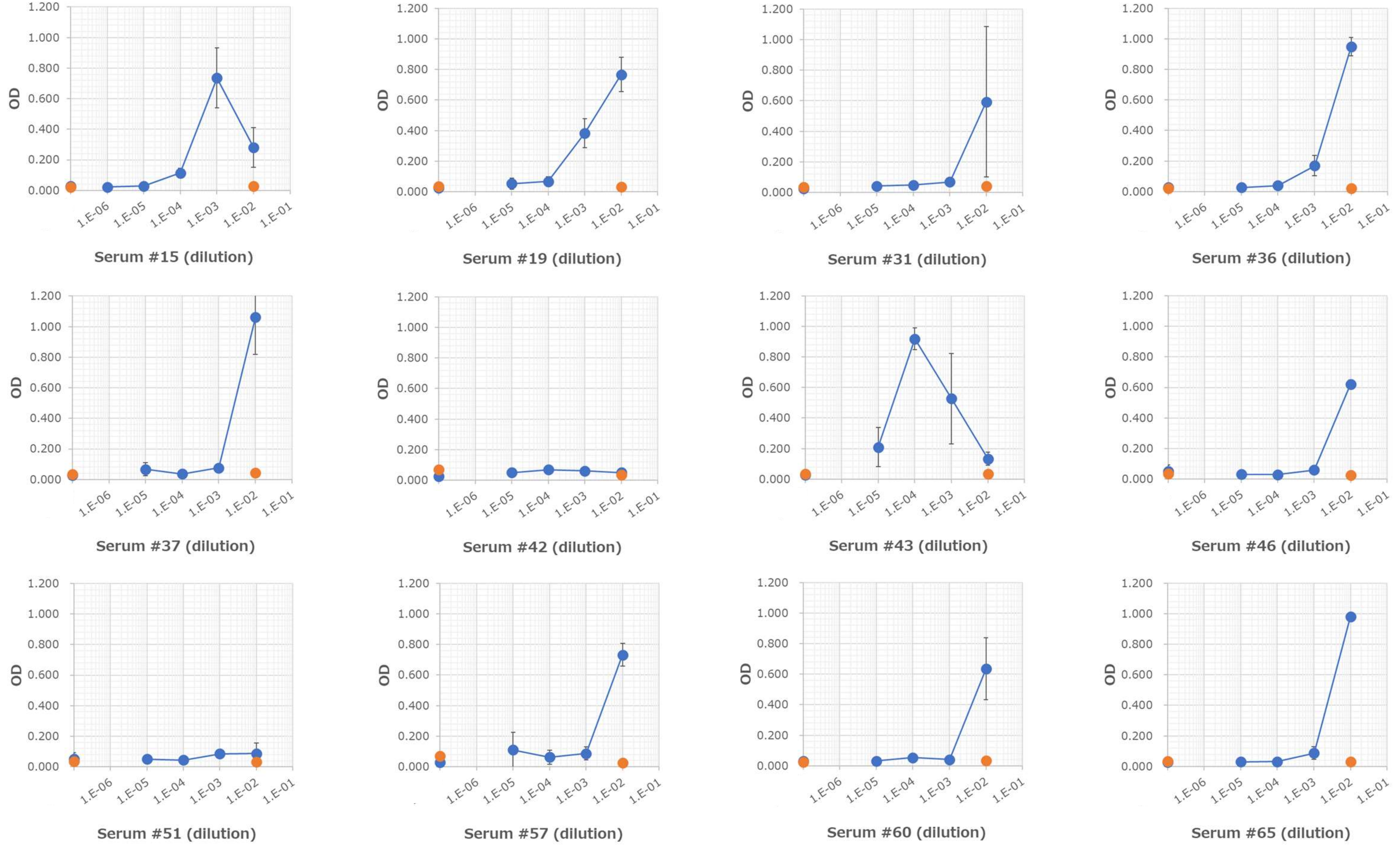

Supplemental Fig. 13-5

(b) Slight ADE

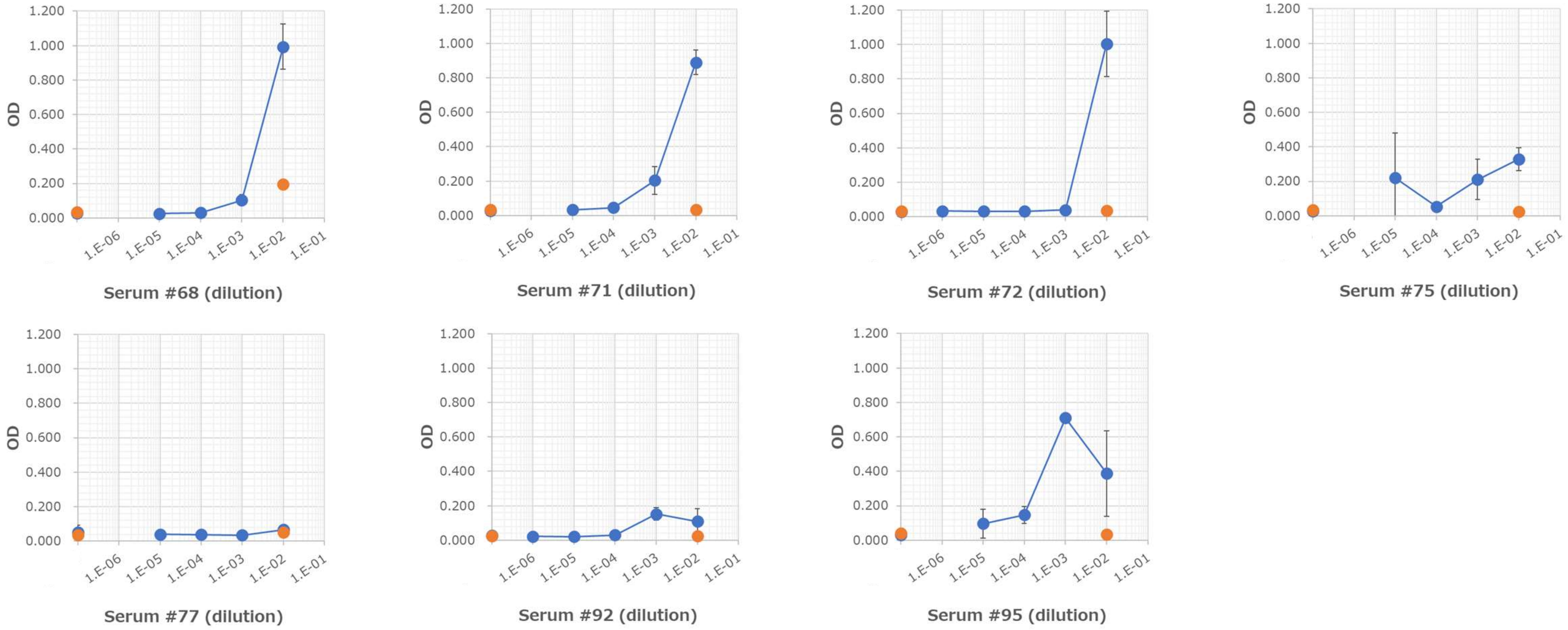

Supplemental Fig. 13-6

(c) No ADE

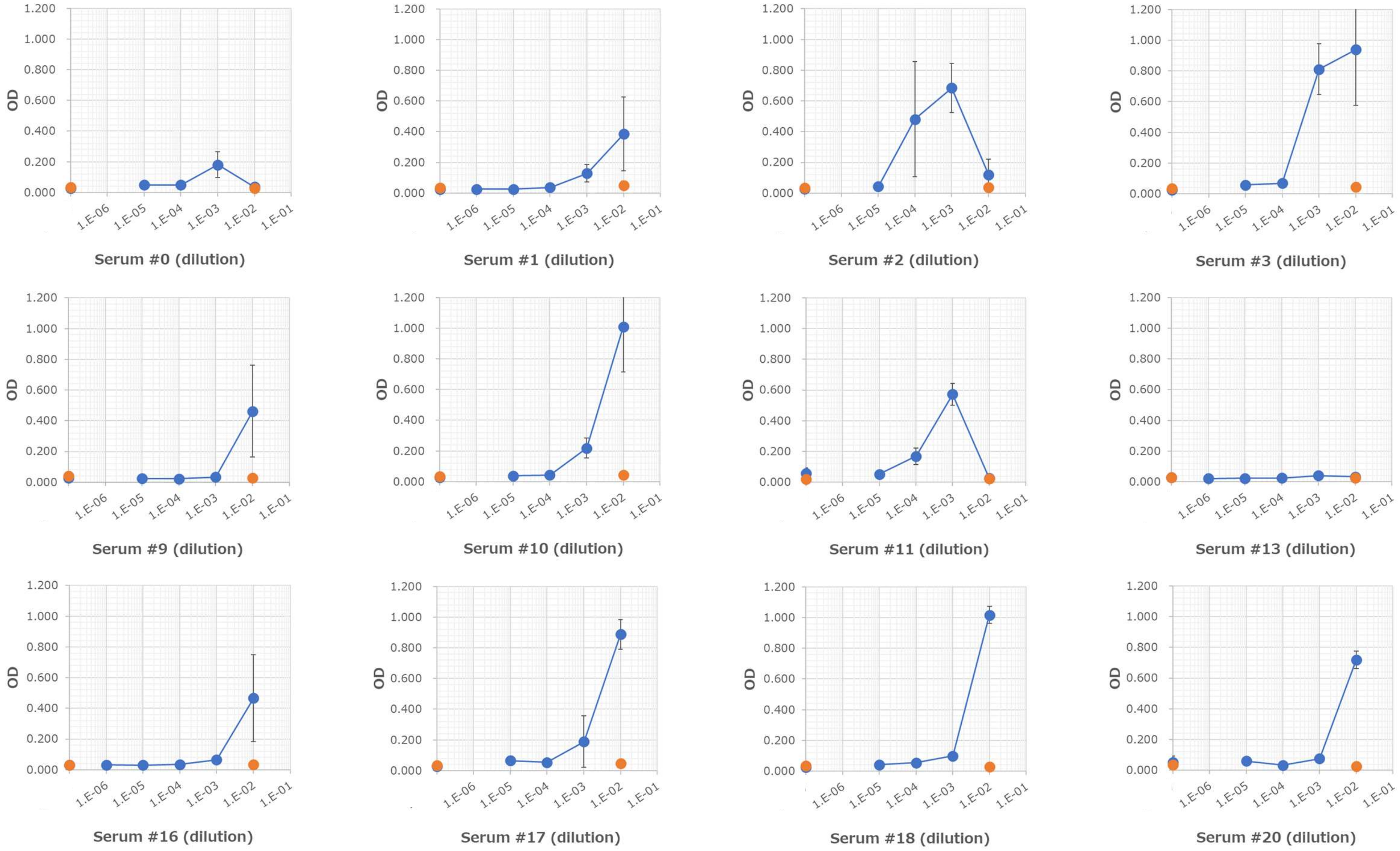

Supplemental Fig. 13-7

(c) No ADE

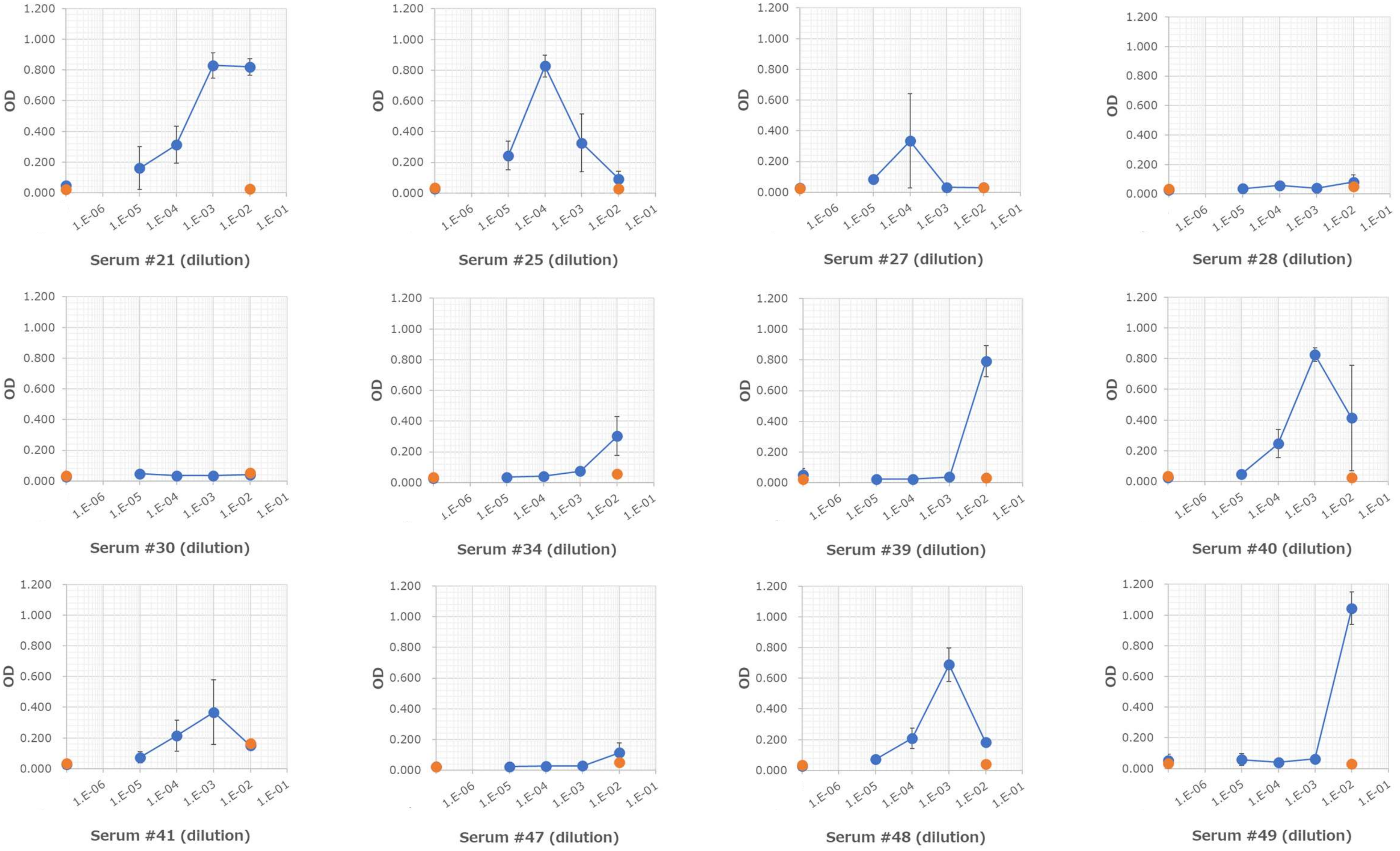

Supplemental Fig. 13-8

(c) No ADE

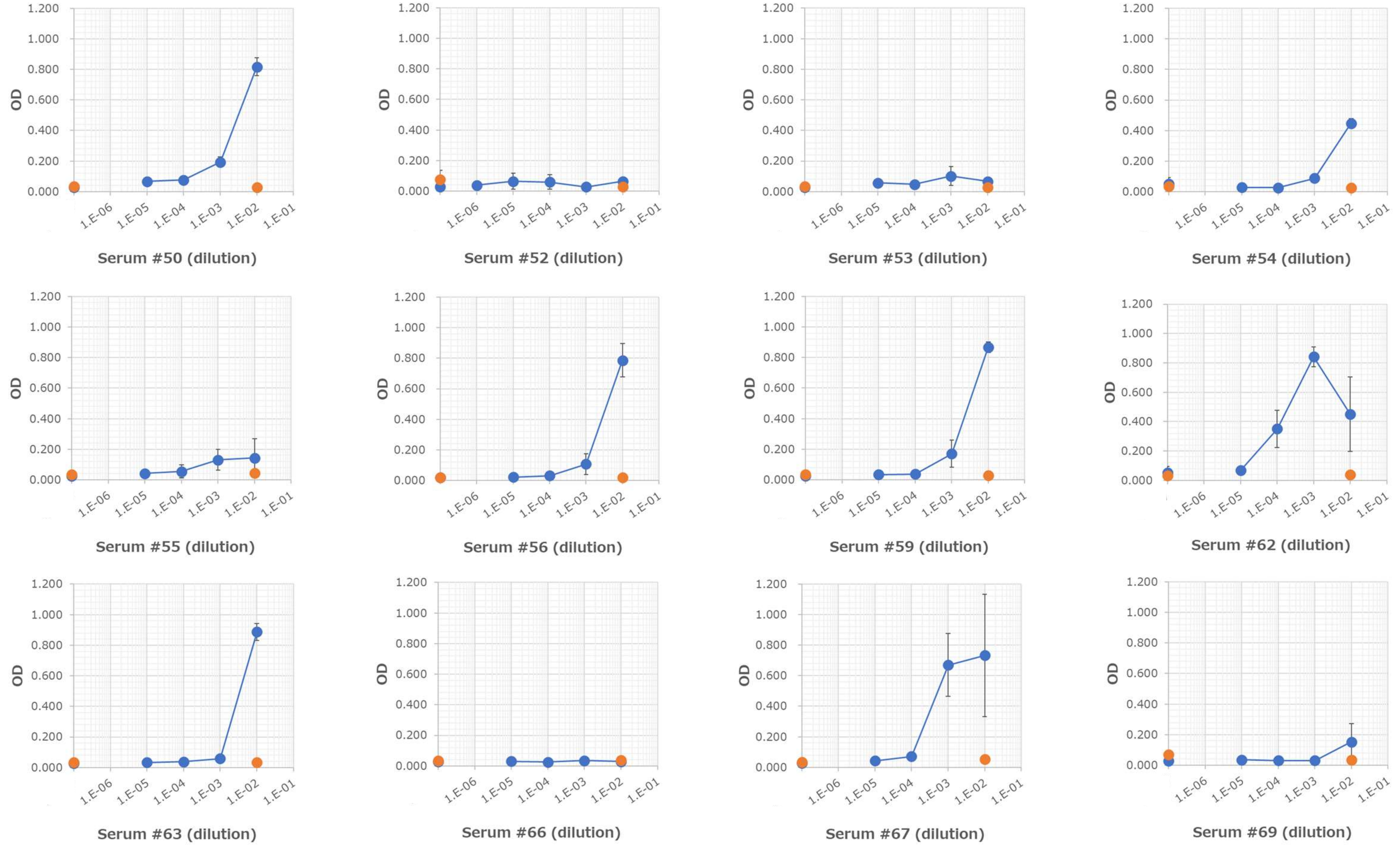

Supplemental Fig. 13-9

(c) No ADE

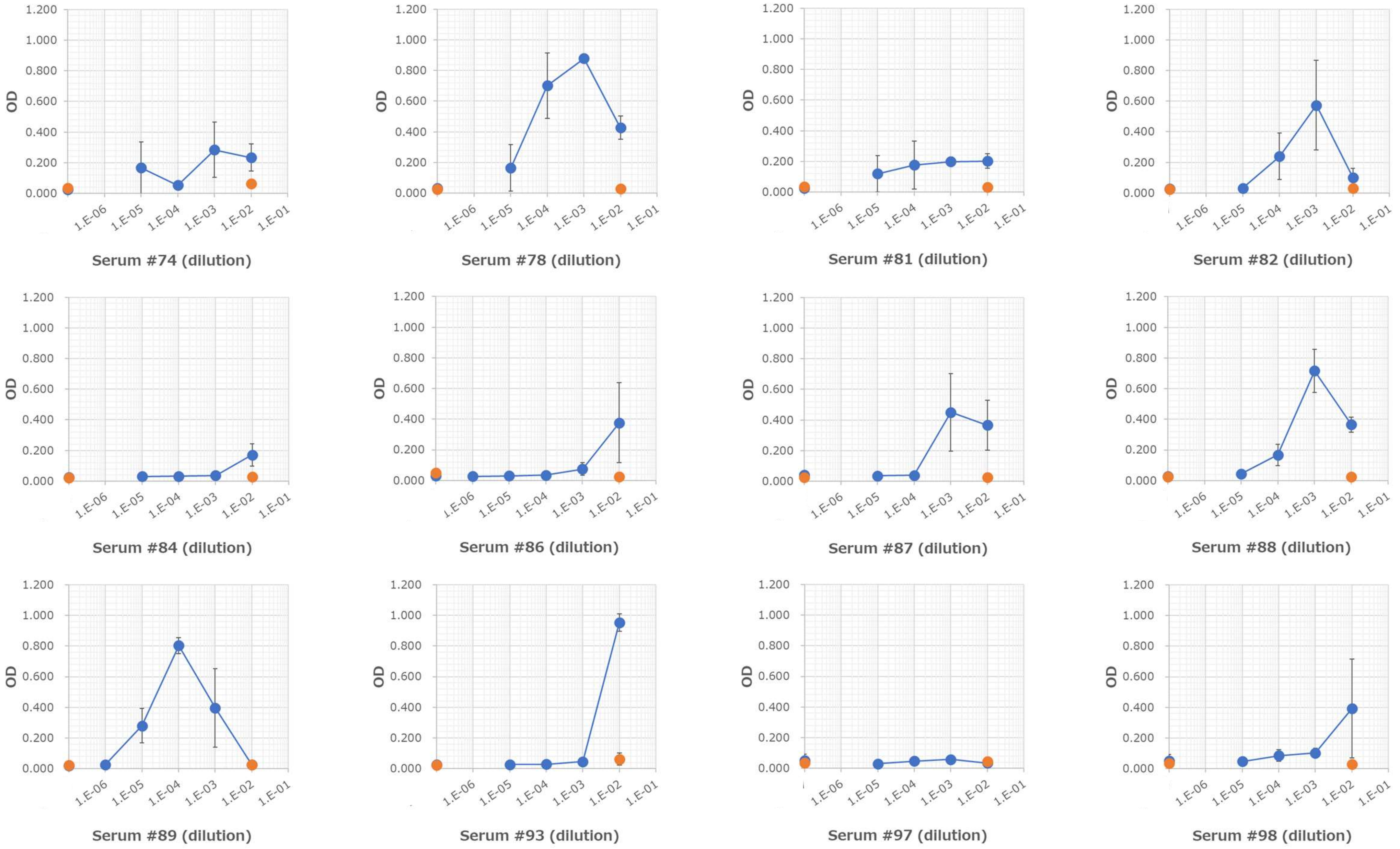

Supplemental Fig. 14

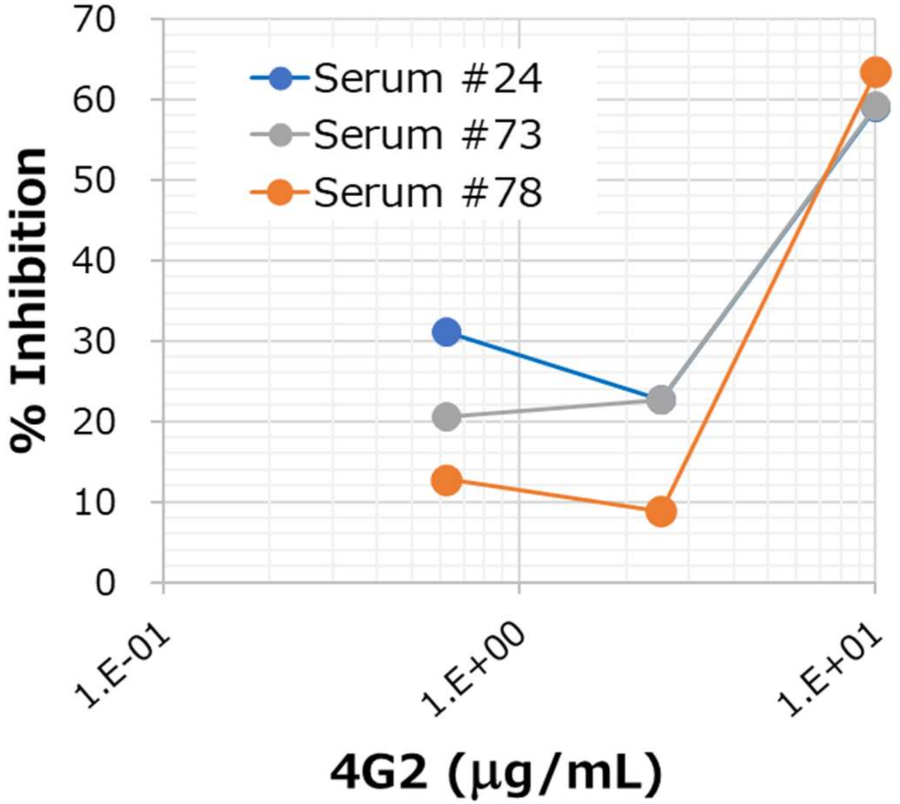

Supplemental Table 1

Information	Age (years)		
	Total	Male	Female
n	100	59	41
Median	72.0	71.0	75.0
(Q1, Q3)	(61.8, 83.0)	(57.5, 80.0)	(68.0, 84.0)

Supplemental Table 2

Anti-SARS-CoV-2 IgG (signal/cut-off ratio)	Group	**Apparent**	**Slight**	**None**
	Median	2.65	2.14	1.95
	(Q1, Q3)	(1.92, 3.08)	(1.87, 2.57)	(1.64, 2.64)
p-value		**Apparent**	**Slight**	**None**
	**Apparent**		0.1779	0.0526
	**Slight**			0.3154

Supplemental Table 3

Experiment	sample			SARS-CoV-2 on day 3 (copies/μL)				Fold increase	
				mean	SD	mean + 3SD	mean + 6SD	**mean + 3SD**	**mean + 6SD**
1	HC6	HC7		152,627	282,615	1,000,472	1,848,317	**6.6**	**12.1**
2	HC4	HC5		336,031	573,933	2,057,831	3,779,631	**6.1**	**11.2**
3	HC1	HC3		374,385	282,229	1,221,071	2,067,757	**3.3**	**5.5**
4	HC2			590,714	237,678	1,303,748	2,016,781	**2.2**	**3.4**
5	#5	#7		147,209	148,385	592,364	1,037,519	**4.0**	**7.0**
6	#22	#29		158,916	107,903	482,625	806,333	**3.0**	**5.1**
7	#35	#45		208,724	131,278	602,557	996,390	**2.9**	**4.8**
8	#26	#43		260,035	153,711	721,167	1,182,299	**2.8**	**4.5**
9	#48	#53		149,480	222,149	815,926	1,482,372	**5.5**	**9.9**
10	#57	#59		1,444,158	2,160,451	7,925,512	14,406,866	**5.5**	**10.0**
11	#69	#76		69,382	66,094	267,663	465,944	**3.9**	**6.7**
12	#4	#24		46,521	32,564	144,211	241,902	**3.1**	**5.2**
13	#73	#77		126,710	121,496	491,197	855,684	**3.9**	**6.8**
14	#49	#63		235,650	326,498	1,215,143	2,194,636	**5.2**	**9.3**
15	#75	#78		394,518	407,108	1,615,843	2,837,168	**4.1**	**7.2**
16	#67	#68		192,638	194,386	775,797	1,358,956	**4.0**	**7.1**
17	#6	#19		191,065	169,326	699,045	1,207,024	**3.7**	**6.3**
18	#99			67,205	75,375	293,330	519,455	**4.4**	**7.7**
19	#58	#66		76,401	99,181	373,945	671,489	**4.9**	**8.8**
20	#38	#44		109,197	112,133	445,596	781,995	**4.1**	**7.2**
21	#36	#37		258,224	210,921	890,987	1,523,749	**3.5**	**5.9**
22	#8	#27		212,340	282,758	1,060,615	1,908,889	**5.0**	**9.0**
23	#46	#64		279,367	179,432	817,664	1,355,961	**2.9**	**4.9**
24	#55	#65		123,599	104,198	436,191	748,784	**3.5**	**6.1**
25	#28	#47		234,120	387,666	1,397,118	2,560,116	**6.0**	**10.9**
26	#89	#97		249,096	294,724	1,133,268	2,017,440	**4.5**	**8.1**
27	#79	#96		14,383	12,381	51,527	88,670	**3.6**	**6.2**
28	#9	#2	#50	1,301,655	1,864,525	6,895,230	12,488,806	**5.3**	**9.6**
29	#60	#70	#72	146,687	118,629	502,575	858,463	**3.4**	**5.9**
30	#3	#61	#88	506,211	886,653	3,166,169	5,826,126	**6.3**	**11.5**
31	#10	#20	#40	501,720	883,060	3,150,899	5,800,078	**6.3**	**11.6**
32	#23	#42	#51	340,349	406,277	1,559,180	2,778,010	**4.6**	**8.2**
33	#90	#93	#95	537,152	814,244	2,979,884	5,422,615	**5.5**	**10.1**
34	#71	#80	#83	237,776	274,806	1,062,195	1,886,614	**4.5**	**7.9**
35	#0	#86	#21	224,665	381,096	1,367,954	2,511,243	**6.1**	**11.2**
36	#30	#62	#82	467,054	941,036	3,290,163	6,113,272	**7.0**	**13.1**
37	#87	#92	#98	147,979	227,297	829,871	1,511,763	**5.6**	**10.2**
38	#85	#91	#94	43,476	74,157	265,947	488,419	**6.1**	**11.2**
39	#1	#11	#16	85,223	229,433	773,523	1,461,822	**9.1**	**17.2**
40	#33	#13	#25	211,076	281,651	1,056,030	1,900,984	**5.0**	**9.0**
41	#14	#15	#31	422,530	676,085	2,450,784	4,479,038	**5.8**	**10.6**
42	#41	#52	#81	215,336	366,334	1,314,339	2,413,342	**6.1**	**11.2**
43	#12	#17	#18	1,509,323	1,178,334	5,044,325	8,579,328	**3.3**	**5.7**
44	#32	#34	#39	306,176	238,325	1,021,152	1,736,129	**3.3**	**5.7**
45	#54	#56	#74	77,881	87,573	340,602	603,322	**4.4**	**7.7**
46	#84			424,000	709,279	2,551,836	4,679,672	**6.0**	**11.0**
